# Systematic review of the osteogenic effect of rare earth nanomaterials and the underlying mechanisms

**DOI:** 10.1186/s12951-024-02442-3

**Published:** 2024-04-16

**Authors:** Ziwei Chen, Xiaohe Zhou, Minhua Mo, Xiaowen Hu, Jia Liu, Liangjiao Chen

**Affiliations:** 1 Department of Orthodontics, School and Hospital of Stomatology, Guangdong Engineering Research Center of Oral Restoration and Reconstruction & Guangzhou Key Laboratory of Basic and Applied Research of Oral Regenerative Medicine, Guangzhou Medical University, Guangzhou, China; 2 Stomatological Hospital, Southern Medical University, Guangzhou, China

**Keywords:** Rare earth nanomaterials, Osteogenesis, Bone regeneration, Angiogenesis, Osteoimmunology

## Abstract

Rare earth nanomaterials (RE NMs), which are based on rare earth elements, have emerged as remarkable biomaterials for use in bone regeneration. The effects of RE NMs on osteogenesis, such as promoting the osteogenic differentiation of mesenchymal stem cells, have been investigated. However, the contributions of the properties of RE NMs to bone regeneration and their interactions with various cell types during osteogenesis have not been reviewed. Here, we review the crucial roles of the physicochemical and biological properties of RE NMs and focus on their osteogenic mechanisms. RE NMs directly promote the proliferation, adhesion, migration, and osteogenic differentiation of mesenchymal stem cells. They also increase collagen secretion and mineralization to accelerate osteogenesis. Furthermore, RE NMs inhibit osteoclast formation and regulate the immune environment by modulating macrophages and promote angiogenesis by inducing hypoxia in endothelial cells. These effects create a microenvironment that is conducive to bone formation. This review will help researchers overcome current limitations to take full advantage of the osteogenic benefits of RE NMs and will suggest a potential approach for further osteogenesis research.

**Graphical abstract:**

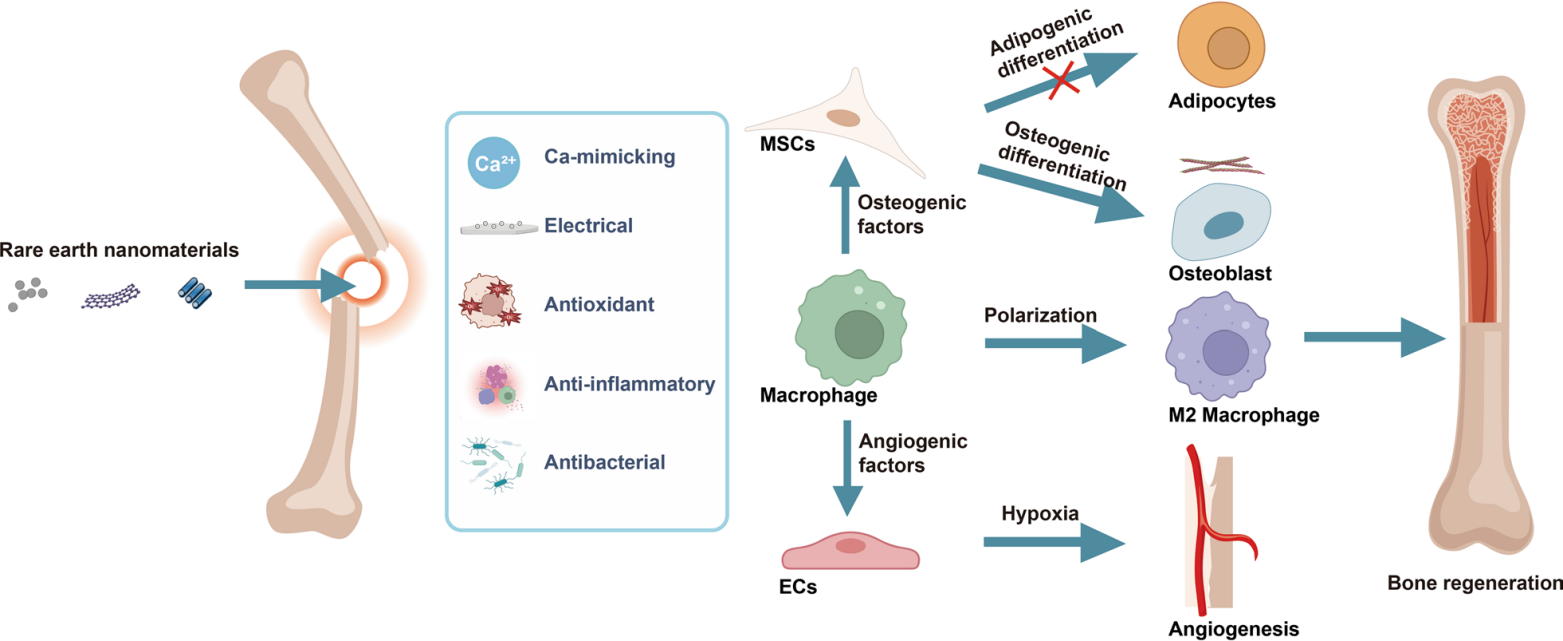

## Background

The inherent self-repair capacity of bone allows fractures or bone defects to heal spontaneously without significant intervention. However, the restoration of extensive bone defects necessitates medical intervention [1]. The use of autografts, an established method for repairing extensive bone defects, is limited by donor scarcity and site morbidity [2, 3]. Therefore, innovative biomaterials with regulatory abilities that can promote bone formation must be explored as substitutes for autografts in tissue repair and regeneration.

**Fig. 1:**
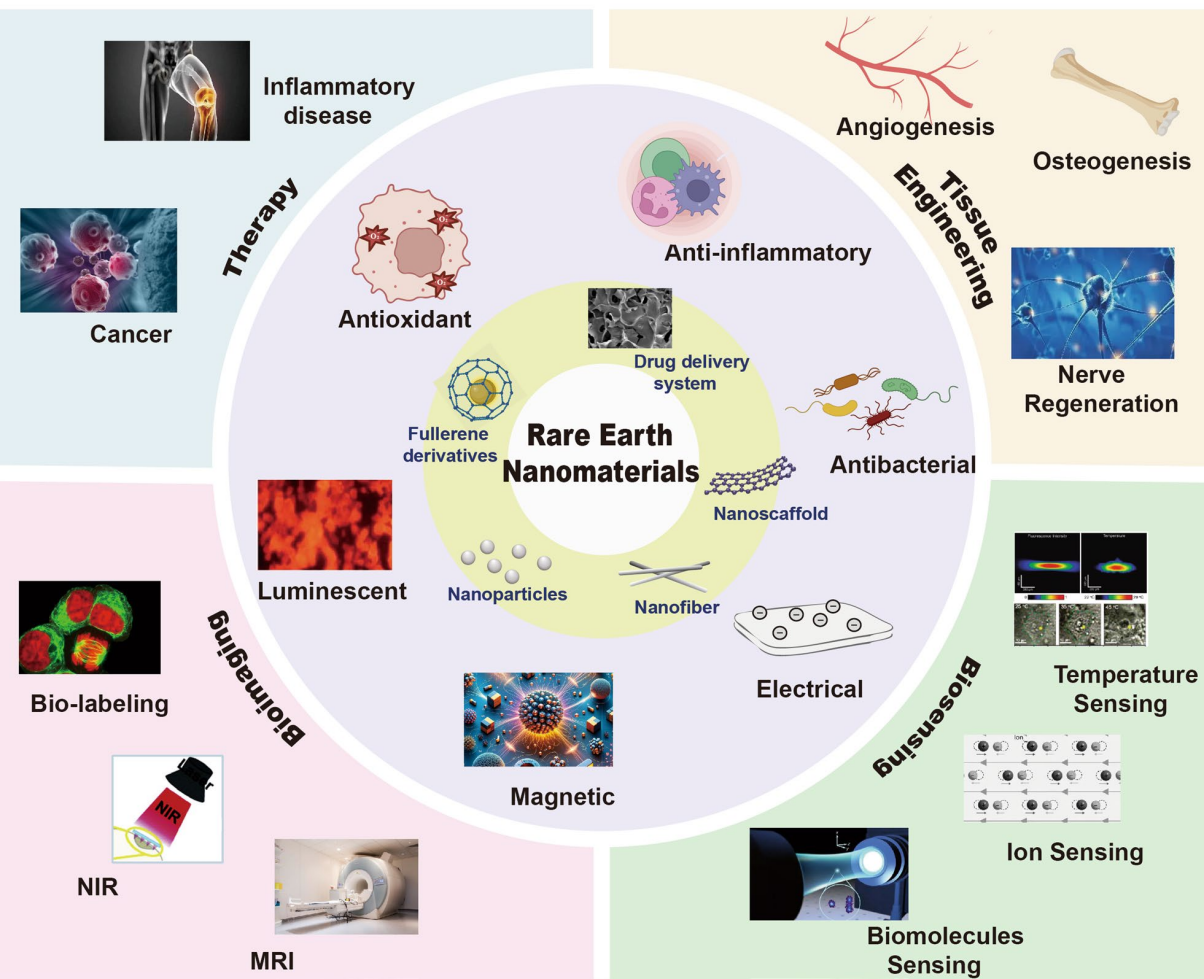
The characteristics and biological applications of RE NMs. RE NMs principally manifest their biological effects through various forms such as nanoparticles, nanofibers, nanoscaffolds, nanoporous drug delivery systems, and fullerene derivatives. They are characterized by their luminescent, magnetic, electrical, antibacterial, anti-inflammatory, and antioxidant properties, which render them extensively applicable in the arenas of therapy, tissue engineering, bioimaging, and biosensing

Rare earth elements (REEs), including cerium (Ce), europium (Eu), lanthanum (La), praseodymium (Pr), neodymium (Nd), samarium (Sa), gadolinium (Gd), terbium (Tb), dysprosium (Dy), holmium (Ho), erbium (Er), thulium (Tm), ytterbium (Yb), lutetium (Lu), yttrium (Y), scandium (Sc) and promethium (Pm) [4], have been extensively investigated for use in the field of bone regeneration due to their flexible redox properties and their unique luminescence and electromagnetic properties [5, 6]. Rare earth nanomaterials (RE NMs) based on REEs have been synthesized through hydrothermal methods [7], freeze-drying technology [8], wet chemical techniques [9], solvothermal methods [10], and other approaches. RE NMs have been investigated and utilized in various biomedical applications, including bone tissue engineering (Fig. 1). For instance, ligand-free NaYF_4_:Yb/Er nanocrystals [11] and NaGdF_4_:Yb/Er nanoparticles [12] have garnered significant attention in the field of bone imaging applications due to their exceptional physicochemical properties for efficient conversion of weak near-infrared light into high-energy visible light. Lanthanum oxide nanoparticles reinforced collagen ƙ-carrageenan hydroxyapatite (HA) biocomposite as an ideal bone filling material, promoting favorable osseointegration [13]. Additionally, RE NMs can be employed in scaffold implantation [14], implant coating [15] and nanofibrous membranes [16]. The high porosity, high specific surface area and oriented structure of RE NMs allow them to effectively accommodate various functional cargoes, including drugs and growth factors, that promote bone formation [4]. The porous structure is also conducive to blood vessel growth, facilitating capillary migration into the bone microenvironment [13].

Previous studies have demonstrated that RE NMs possess exceptional osteogenic properties both in vitro and in vivo [17–20]. Cerium oxide nanoparticles (CeO NPs) emerged as one of the first RE NMs in medical applications, as Ce is the most abundant REE. Moreover, due to their remarkable antioxidant, anti-inflammatory, antibacterial, angiogenic, and antiapoptotic activities, CeO NPs have attracted significant attention for use in bone regeneration [21]. With further investigation, additional RE NMs, such as lanthanum oxide NPs [13], Gd@C_82_(OH)_22_ NPs [22] and β-NaGdF_4_:Yb/Er upconversion NPs [12], have been found to promote osteogenesis. RE NMs not only regulate the functions of mesenchymal stem cells (MSCs) and osteoblasts but also promote bone formation by modulating the immune environment [23] and promoting angiogenesis [24]. However, how their inherent properties affect bone regeneration and the possible common osteogenic mechanisms involved have not been reviewed in detail.

This review presents an overview of the physicochemical properties and biological advantages of RE NMs as osteogenic materials, with particular emphasis on their capacity to regulate cellular function through multiple molecular mechanisms to promote osteogenesis. Moreover, we elucidate how RE NMs modulate macrophage differentiation and polarization to promote bone regeneration. Their regulation of endothelial hypoxia to modulate angiogenic–osteogenic coupling is also discussed. Finally, we summarize the crucial factors that influence the osteogenic effects of RE NMs. This review can serve as a valuable reference for studying the role of RE NMs in bone formation.

## Physicochemical properties and biological advantages of RE NMs

### Physicochemical properties of RE NMs

RE NMs possess unique physicochemical properties, including calcium (Ca)-mimicking and electrical characteristics, that endow them with osteogenic potential. These properties enable them to directly replace Ca in HA, contributing to bone deposition and activating calcium channels to promote bone formation. The excellent piezoelectricity and conductivity of these materials also allow them to accurately mimic natural bone, facilitating the repair of bone defects.

#### Calcium-mimicking properties

RE NMs can release small amounts of RE ions during their slow degradation [8]. After internalization by cells, RE NMs localize to mitochondria [25], lysosomes [26] and the endoplasmic reticulum [27] and are abundant in both the cytoplasm [11] and the nucleus [27]. The fraction of RE NMs localized within lysosomes undergoes acidification since their ionolysis kinetics are dependent on the pH [28]. Y_2_O_3_ NPs, for instance, localize to acidifying intracellular lysosomes after they are taken up by BMSCs, and they undergo dissolution and transformation from Y_2_O_3_ NPs to Y^3+^ [29]. Most RE ions have biological properties similar to those of Ca^2+^ and exhibit the ability to structurally or functionally replace Ca^2+^ to exert positive or negative effects on bone regeneration [30].

The ionic radii of RE ions range from 0.0848 nm (Lu) to 0.1034 nm (Ce), which is similar to the Ca radius of 0.104 nm [31]. This implies that RE ions can substitute for Ca^2+^ in HA, thereby increasing its physical and chemical stability in bones [32]. When RE ions interact with cells, they can activate Ca^2+^ receptors such as calcium-sensitive receptors (CaRs), increasing intracellular Ca^2+^ levels and promoting osteogenic differentiation [33]. However, the competitive binding of RE ions and Ca^2+^ can partially block Ca^2+^ channels. For example, RE ions block stretch-activated calcium channels (SACCs) [34] and voltage-gated calcium channels (VGCCs) [35], thereby impeding the modulation of bone and cartilage function by Ca^2+^ [36, 37].

The potential positive or negative effects of the mimicry of Ca^2+^ by RE ions have not been fully specified, as the effects of RE ions binding to different Ca^2+^ receptors can vary. For instance, Tb^3+^-bound cadherins exhibit a more elongated and less curved structure, resulting in the inhibition of cell adhesion [38]. Calmodulin binding sites undergo conformational and dynamic changes upon binding to RE ions (Tb^3+^, La^3+^, and Lu^3+^) [39], potentially leading to osteoblast growth and differentiation [40]. Therefore, understanding the biological effects of RE ions on Ca^2+^-binding proteins and Ca^2+^ channels is crucial for elucidating the physiological implications of the substitution of RE ions for Ca^2+^.

#### Electrical properties

The electron configurations of [Xe]4fn (n = 0–14) and the abundant unpaired electrons in RE ions endow them with high electronic energy levels and long-lasting excitation states, making them electrically active [41]. RE NMs exhibit diverse electrical properties, enabling broad applications in various fields [42], such as electronic transducers [43], ultrahigh-temperature electromechanical engineering, ultrasensitive probes [44] and bone regeneration [45].

Natural bone is an electrosensitive tissue. When physiological compressive loads are applied to the bone, it generates negative charges through piezoelectric potential. These negative charges effectively stimulate VGCC and SACC, leading to an increase in intracellular Ca^2+^ levels and promoting bone regeneration [46]. Due to the piezoelectric properties of RE materials, the use of RE ions as dopants can increase the piezoelectricity of a material. The incorporation of Eu [47] and Sm [48] dopants into the PMN-PT ceramic system, for instance, increases piezoelectricity. RE doping generates electrostatic interactions (such as H-bonding) [49] and stabilizes the crystal structure of NPs, which decreases their dielectric constant, in turn facilitating the distribution of polarized electric fields on the NP surface for increased composite piezoelectricity [45].

In some cases, RE doping increases electrical conductivity, thereby simulating the cellular microenvironment and promoting normal cell growth [50]. The presence of oxygen vacancies endows RE NMs with significant potential [51] for increasing the electronic conductivity and charge mobility rate of materials [52], which facilitates the restoration of electrical current in bone

### Biological advantages of RE NMs

#### Antioxidant activities

Bone defects are usually accompanied by local microvascular rupture, inflammatory injury and infection, which poses a challenge for bone regeneration in anoxic microenvironments [53]. Tissue hypoxia can lead to the production of reactive oxygen species (ROS), which primarily arise as byproducts of electron leakage from the mitochondrial electron transport chain [54] and nicotinamide adenine dinucleotide phosphate (NADPH) oxidase (NOX) [55]. A high concentration of ROS can induce osteoblast death and thus interfere with the osteogenic differentiation of BMSCs and osteoblast precursor MC3T3-E1 cells [53]. Therefore, the removal of excess ROS is highly important for promoting bone regeneration.

The antioxidant activity of RE NMs, such as Ce- [56], La- [57], Gd- [58, 59], Y- [59, 60], Eu- [61], Yb- [62] and Er-based [62] nanomaterials, can counteract the oxidative damage caused by ROS. RE NMs are used as antioxidants in treating diseases such as diabetes [60], hepatic failure [63], and neurodegenerative diseases [64]. CeO NPs [16], Gd@C_82_(OH)_22_ [65] and Y_2_O_3_ NPs [66] can effectively remove intracellular ROS in bone cells, promoting cell proliferation and osteogenic differentiation and thereby facilitating bone regeneration.

The antioxidant mechanisms of RE NMs primarily involve their enzyme-like characteristics, generation of oxygen vacancies, and activation of relevant signalling pathways to increase the expression of antioxidant enzymes. (i) Enzyme-like characteristics. CeO NPs are the RE NMs most widely used for promoting bone regeneration. One of the key reasons for their popularity is their excellent enzyme-mimicking activities, which make them highly stable and cost-effective alternatives to natural enzymes. These enzymes can mimic superoxide dismutase (SOD), catalase (CAT), oxidase and peroxidase, phosphatase, DNase I and urease [67]. The presence and switching of Ce^3+^ and Ce^4+^ mixed valence states, along with the presence of oxygen vacancies in CeO, are the crucial factors in its enzyme-like characteristics [68]. SOD-like activity was dominant in CeO with a high Ce^3+^/Ce^4+^ ratio [69], which is pivotal in the clearance of ROS [70]. This activity was found to be highly dependent on pH, and CeO was shown to act as an oxidase instead of a peroxidase at acidic pH [68]. (ii) The generation of oxygen vacancies. Oxygen vacancies refer to the vacancies that occur in metal oxides when oxygen detaches from the lattice; these vacancies can reduce compounds and are considered to be valuable tools for eliminating ROS [67]. In Eu-doped yttrium oxide (Y_2_O_3_) [71] and Eu-doped lutetium oxide (Lu_2_O_3_) [72], REE doping alters the lattice constant, increasing the number of oxygen vacancies. Oxygen vacancies are an inherent defect in the crystal structure of CeO NPs [56] due to the imbalance between Ce^3+^ and Ce^4+^. (iii) Upregulated expression of antioxidant enzymes, including superoxide dismutase (SOD), catalase (CAT), glutathione-s-transferase (GST) and hemeoxygenase-1 (HO-1). RE NMs may upregulate the expression of antioxidant enzymes through the FOXO1 pathway [73], the PI3K-AKT-mammalian target of rapamycin (mTOR) pathway, the ERK-MEK signalling pathway [74] and the nuclear factor erythroid 2-related factor 2 (Nrf2)-antioxidant response element (ARE) pathway [75].

#### Anti-inflammatory activities

The process of bone regeneration involves three sequential and overlapping phases: inflammation, regeneration, and remodelling. In a normal bone repair scenario, inflammation is initiated immediately after injury and promptly resolved. However, persistent acute or chronic inflammation can impede the healing and regeneration of bones [76, 77]. Therefore, resolving inflammation following the proinflammatory phase could be an effective therapeutic strategy for enhancing bone regeneration.

Previous studies have shown that Ce- [78], Y- [60, 79], La- [23], and Gd-based [80] nanomaterials regulate the immune response and reduce inflammation. CeO NPs [9, 75] and magnetic lanthanum-doped hydroxyapatite/chitosan (MLaHA/CS) nanoscaffolds [23] have been reported to reduce persistent inflammation and accelerate the transition to the bone repair phase.

The anti-inflammatory mechanism of RE NMs is mainly attributed to promoting macrophage M2 polarization [8], as observed in Ce [81, 82], Gd [83], La [84] and Nd:YAG laser irradiation [85, 86]. M2 macrophage polarization is conducive to the regression of inflammation and the stability of bone repair and directly inhibits the production of inflammatory mediators (*e.g.,* TNF-α, IL-6 and iNOS) [87, 88]. For example, CeO NPs may induce the expression of arginase (Arg) [89], which competes with the inflammatory mediator iNOS for its substrates [90], thereby reducing inflammation in J774a [87]. Immunomodulatory biomaterials have the potential to modulate inflammation and promote bone healing. We will discuss this prospect in more detail below.

#### Antibacterial activities

When bacterial activity occurs in a bone defect, the regenerative capacity of the bone can be severely compromised, leading to open comminuted fractures or to the development of severe osteomyelitis [91]. Allografting [92] and artificial bone [93] have been utilized for treating bone defects, but they may present challenges such as infection. Therefore, developing a biomaterial that effectively prevents bacterial adhesion and proliferation is crucial for promoting bone regeneration. *Wakabayashi* [94] demonstrated that RE ions exhibit antibacterial activity against *Staphylococcus aureus* and *Escherichia coli* (*E. coli*)*.* It has been reported that Ce- [95], La- [96, 97], Y- [98], Tb- [99], Dy- [100], Nd- [101, 102], Yb- [103], Sm- [104] and Ho-based [105] nanomaterials possess extensive antibacterial properties and high biocompatibility, making them promising candidates for bone regeneration [106].

The antibacterial mechanisms of these materials include the following: (i) nanoscale surface topography induces chemical reactions at interfaces that repel bacterial cells, hindering biofilm formation and resisting adhesion [107]. (ii) RE NMs directly cause membrane damage [95] or change the morphology of the bacterial membrane [108], decreasing cell viability. For example, CeO NPs [95] physically penetrate the membrane and destroy the integrity of the *E. coli* bacterial membrane, leading to the death of *E. coli.* iii) Some RE NMs, such as terbium oxide nanoparticles (Tb_4_O_7_ NPs) [99], can disable bacteria by inducing oxidative stress. This might occur because the antioxidant and oxidative activities of these materials are pH dependent, similar to those of CeO NPs. The proton motive force decreases the local pH (as low as 3.0) in the cytoplasm and membrane of bacterial cells [109, 110]. The antioxidant activity of these materials is thus transformed into oxidative activity under acidic conditions [111]. CeO NPs [112] may exert the same antibacterial effects.

## Effects of RE NMs on osteogenesis and the underlying mechanisms

**Fig. 2:**
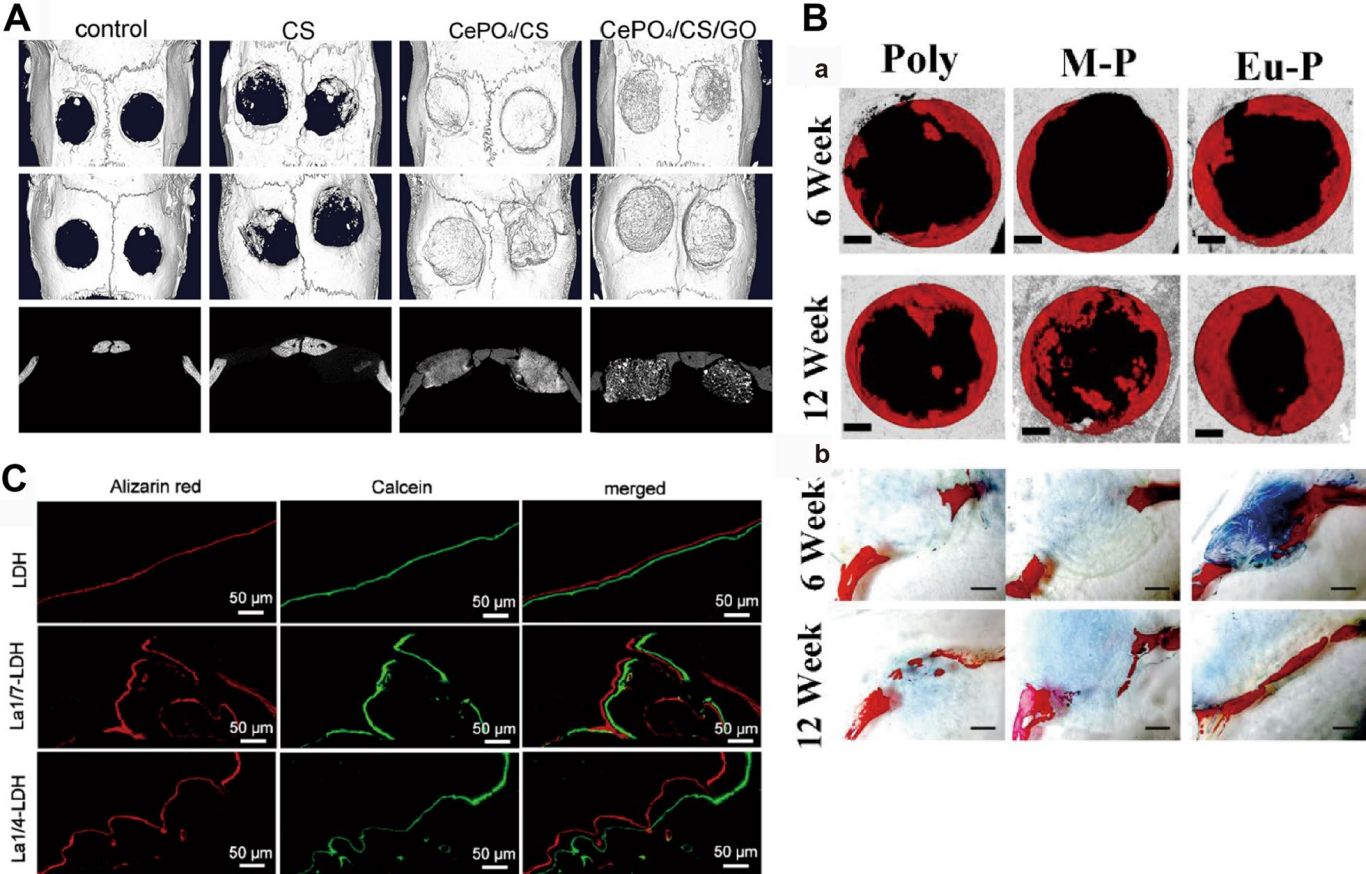
RE NMs promote osteogenesis in vivo. **A** Micro‑CT images of skulls from the control, CS, CePO_4_/CS, and CePO_4_/CS/GO groups 3 months after surgery. Source: Reprinted with permission from ref. [8]. **B** Effect of Eu-MSNs on osteogenesis in vivo. Representative micro-CT images of new bone formation (the grey background represents a normal skull, the black holes represent the surgically created 5 mm diameter cranial defect, and the red represent the newly formed bone at the defect site, according to analysis by CTAn software for Micro-CT. (a) Corresponding statistical analysis. (b) VG staining of the cranial defects shows that more new bone (red) was formed at the cross section of the defect in the Eu-P groups at 6 and 12 weeks. Source: Reprinted with permission from ref. [175]. Copyright 2024, with permission from Elsevier. **C** Fluorochrome-labelling analysis of bone mineralization by calcein (green) in La/LDH at 14 days and alizarin red (red) at 7 days before euthanasia. Source: Reprinted with permission from ref. [14]. CS, chitosan; GO, Graphene oxide; Eu-MSNs, europium-doped mesoporous silica nanospheres; Poly, polymer; M-P, MSNs coated polymer film; Eu-P, Eu-MSNs coated polymer film; La/LDH, lanthanum-substituted MgAl layered double hydroxide

Due to the unique physicochemical and biological properties of RE NMs, they have been extensively investigated as osteogenic materials. RE NMs induce a faster healing with regeneration of lost bone tissue in vivo (Fig. 2). For instance, La-LDH nanohybrid scaffolds increase the bone mineral density (BMD) and the ratio of bone volume to tissue volume (BV/TV) after 12 weeks of implantation in rat cranial defect model [14]. Eu-doped Gd_2_O_3_ nanotubes extremely increased the maximal load of bones [113]. These are attributed to their excellent osteogenic properties as we discussed in Part 2.

The calcium-mimicking of RE NMs enable them to replace Ca in bones and improve BMD in vivo. Additionally, they activate Ca^2+^ channels in MSCs to regulate intracellular Ca^2+^ levels and facilitate osteogenic differentiation by simulating Ca^2+^ and affecting electric fields. The antioxidant activities of RE NMs can safeguard cells and the osteogenic microenvironment against ROS-induced damage. Furthermore, their anti-inflammatory and antibacterial properties effectively alleviate inflammation and bacterial infections at bone defects and surrounding implants, thus expediting bone repair. Studies have demonstrated the ability of RE NMs to promote osteogenesis, as shown in Table 1. We will detail the osteogenic mechanism of RE NMs and explore the unreported RE NMs that may have osteogenic effects.

**Table 1:** Details of direct osteogenic effects of RE NMs

RE NMs	Synthetic method	Physicochemical properties and biological advantages	Model	Mechanism	Osteogenic effect	Refs.
CeO NPs	Microemulsion method Wet chemical technique	Antioxidative Anti-inflammatory Antibacterial activities	BMSCs Mouse midfemoral defect model	DHX15/p38 MAPK	Promote cell proliferation and hypertrophic differentiation Promote ectopic osteogenesis through endochondral ossification	[114]
			MC3T3-E1 cells	Fam53B/Wnt/β-catenin	Promote cell proliferation, osteogenic differentiation and mineralization	[139]
			hBMSCs RAW 264.7 cells	Modulates inflammation microenvironment		[9]
			hBMSCs	Autophagy	Promote osteogenic differentiation and mineralization Protect against ionizing radiation induced cellular damage	[54]
Nano-shaped CeO coating	Hydrothermally method	Antioxidative Anti-inflammatory activities	MC3T3-E1 cells RAW264.7 cells HUVECs Rat femoral condyles	Scavenge ROS	Promote cell adhesion, proliferation osteogenic differentiation and mineralization Promote M2 polarization of macrophages and H typed blood vessel formation	[15]
CePO4/CS/GO scaffold	Hydrothermal method and freeze-drying technology	Anti-inflammatory activities	MC3T3-E1 cells RAW264.7 cells	BMP2/Smad1/5	Promote cell adhesion, migration, osteogenic differentiation and mineralization Promote angiogenesis and M2 polarization of macrophages	[8]
Ce-BG scaffolds	Freeze-drying technology	/	hBMSCs Rat cranial defects	ERK1/2	Promote cell proliferation, osteogenic differentiation and collagen deposition Promote bone regeneration	[127]
nCe-scaffold	/	Antioxidant activities	MSCs Rat calvarium defect model	Integrin/TGF-β co-signaling	Promote osteogenic differentiation and mineralization Promote new bone formation	[133]
nCeHA/CS	/	/	hBMSC MC3T3-E1 cells BMMs Rat calvarium defect model	BMP2/Smad5 RANK/RANKL/OPG	Promote cell adhesion, proliferation, migration, osteogenic differentiation Inhibiting osteoclast differentiation Decreased bone resorption	[164]
Ce-MBGNs	Microemulsion-assisted sol–gel method	Anti-inflammatory Antioxidative activities	L929 cells J774a.1 cells SAOS-2 cells	RANK/RANKL/ OPG	Promote cell adhesion, proliferation, migration, osteogenic differentiation and mineralization Inhibiting osteoclast differentiation	[78]
Eu-Gd_2_O_3_ Nanotubes	Coprecipitation process	/	MC3T3 cells Mouse oral model	BMP/Smad 1/5	Promote cell proliferation, osteogenic differentiation and mineralization Enhance the bone mineral density and bone biomechanics	[113]
Eu-MSNs	A one-pot method	/	BMSCs HUVECs	Immune response of macrophages	Promote osteogenesis and angiogenesis	[175]
Gd-BTO NPs	Hydrothermal method	Electrical properties	MC3T3 cells	Calcineurin/NFAT	Promote osteogenic differentiation	[145]
[Gd@C_82_(OH)_22_] n nanoparticles	Soft-template (CTAB) method	Anti-inflammatory Antioxidative activities	hMSCs	Modulates inflammation-induced osteogenesis through JNK/STAT3	Protect cell viability, promote osteogenic differentiation in inflammatory microenvironment	[65]
			MSCs Ovariectomized rats	BMP/Smad1/5	Promote osteogenic differentiation and mineralized nodule formation Inhibit adipogenic differentiation	[22]
GdPO_4_/CS/Fe_3_O_4_ scaffolds	hydrothermal method	Anti-inflammatory activities	hBMSCs MC3T3-E1 cells RAW264.7 cells	BMP2/Smad	Promote cell proliferation and osteogenic differentiation Promote M2 polarization of macrophages	[7]
La-LDH nanohybrid scaffolds	Coprecipitation And freeze-drying technology	/	rBMSCs-OVX BMMs Calvarial bone defect OVX rats model	Wnt/β-catenin	Promote cell proliferation and osteogenic differentiation	[14]
La_2_O_3_ NPs	Sol–gel method	/	EA. hy926 MG-63 Rat tibia defects	/	Promote EA. hy926 proliferation and angiogenesis Promote OPN、OCN expression Promote bone defect repair	[13]
Tb/MBG nanospheres	Sol–gel method	/	MC3T3 cells	/	Promote hydroxyapatite-mineralization in vitro	[201]
Tb/Eu–FHA nanorods	Hydrothermal method	/	BMSCs	/	Promote osteogenic differentiation and COL1a1 production	[129]
Y_2_O_3_ NPs-PCL	Precipitation with ammonium hydroxide	Antioxidative activities	UMR-106 cells	/	Promote cell proliferation Promote neovascularization	[66]
NaGdF_4_:Yb/Er NPs	Thermal co-precipitation process	/	rBMSCs	/	Promote cell proliferation, osteogenic differentiation and mineralization Inhibit adipogenic differentiation	[12]
NaYF_4_: Yb/Er nanocrystals	/	/	rBMSCs	/	Promote cell proliferation, osteogenic differentiation and mineralization Inhibit adipogenic differentiation	[11]

Bone marrow macrophages (BMMs); Human Mesenchymal Stem Cells (hMSCs); Graphene-modified CePO_4_ nanorods (CePO_4_/CS/GO scaffold:); Cerium containing mesoporous bioactive glass nanoparticles (Ce-MBGNs); Cerium oxide nanoparticles (CeO NPs); Cerium oxide nanoparticles-modified bioglass (Ce-BG); Mesenchymal stem cells (MSCs); Europium-Doped Gd_2_O_3_ (Eu-Gd_2_O_3_); Gadolinium-doped barium titanate nanoparticles (Gd-BTO NPs);(Poly(lactic-co-glycolic acid)) polymer (PBLG); Lanthanum-substituted MgAl layered double hydroxide (La-LDH); Lanthanum Oxide Nanoparticles(La_2_O_3_ NPs); Mesoporous bioactive glass nanofibers (MBG); nacre-mimetic cerium-doped layered nano-hydroxyapatite/chitosan layered composite scaffold (nCeHA/CS); Terbium (Tb) doped mesoporous bioactive glasses (Tb/MBG); Terbium (Tb) or europium (Eu)-doped fluorapatite nanorods (Tb/Eu–FHA); Yttrium oxide nanoparticles incorporated in polycaprolactone (Y_2_O_3_ NPs-PCL)

### Direct osteogenic effects and mechanisms

#### Promotion of cell proliferation, adhesion and migration

The ability of materials to promote cell proliferation, adhesion and migration largely reflects the interaction between the materials and the cells. The stable adhesion and proliferation of MSCs and osteoblasts on a biomaterial surface are prerequisites for the promotion of bone repair and bone integration. Subsequently, the cells adhering to the material surface migrate and anchor to the site of bone defects, where they perform osteogenic functions [3].

Studies have reported that RE NMs, including Ce [16, 114], Gd [10, 22, 115], Eu [116], La [13], Y [66], NaGdF_4_:Yb/Er NPs [12] and NaYF_4_:Yb/Er NPs [11], have high biocompatibility and can promote the proliferation of MSCs, preosteoblasts and osteoblasts. RE NMs have been shown to accelerate cell cycle progression [117] and promote mitotic spindle formation [12]. The interactions of RE NMs with classical osteogenic signalling pathways, such as the BMP/Smad [113] and Wnt/β-catenin [14] pathways, as well as the activation of Ca^2+^-related pathways through Ca^2+^ substitutes, are believed to play pivotal roles in this process. For instance, Gd^3+^ can stimulate CaRs and increase intracellular Ca^2+^, thereby promoting mitogenic responses in MC3T3-E1 cells [33]. This partially elucidates the mechanism underlying the promotion of cell proliferation by Gd-based nanomaterials. Additionally, the antioxidant properties of RE NMs, such as Gd@C_82_(OH)_22_ [65] and Y_2_O_3_ NPs [66], can reduce intracellular ROS production, relieve oxidative stress, and increase the viability and proliferation of osteoblasts. Their antibacterial activity can also effectively mitigate the cell damage and death caused by bacterial-triggered ROS production, thereby promoting cell function [118].

RE NMs regulate cell adhesion and migration through modulating the cytoskeleton. (i) The formation of filopods and pseudopods, such as CeO NPs [114] and La-substituted layered double hydroxide (La-LDH) nanohybrid scaffolds [14] occurs in advance of the cell movement, where long f-actin molecules within the cell protrude through the extended front line to sense nanotopographical cues and determine the direction of migration [119]. This process is followed by the formation of focal adhesions (FAs) in front of the cell: the FAs are formed by f-actin and the ECM and provide tension to move the cell forward under the action of stress fibers [120]. (ii) The overall diffusion of actin is increased, and the diffusion area of MSCs is expanded [18, 54], facilitating the generation of abundant FAs and promoting rapid cytoskeletal rearrangement, thus accelerating the migration process.

The three‑dimensional (3D) interconnected macropores with pore sizes of 100–200 μm of La-LDH nanohybrid scaffolds facilitated the adhesion and pseudopodium migration of rBMSCs-OVX along the pore walls and promote the in-growth of the newly formed bone tissues from the surfaces into the interiors [14]. This may be due to the fact that the nanoscale porous structures of RE NMs deliver mechanical signals to cells via integrins [121] and Rho GTPases [122] signalling pathways, thereby controlling cytoskeletal reorganization and promoting cell adhesion and migration. The orientation of the nanofibers can also directionally regulate the direction of cell migration [123]. Moreover, Ce^3+^ upregulates stromal cell-derived factor-1 (SDF-1) mRNA expression (124), which plays a crucial role in the BMP2-induced recruitment, migration, and osteogenic differentiation of BMSCs [125]. These findings suggest that Ce-based nanomaterials promote cell migration partly through activation of the BMP signalling pathway and upregulation of the expression of SDF-1.

#### Promotion of osteogenic differentiation

**Fig. 3:**
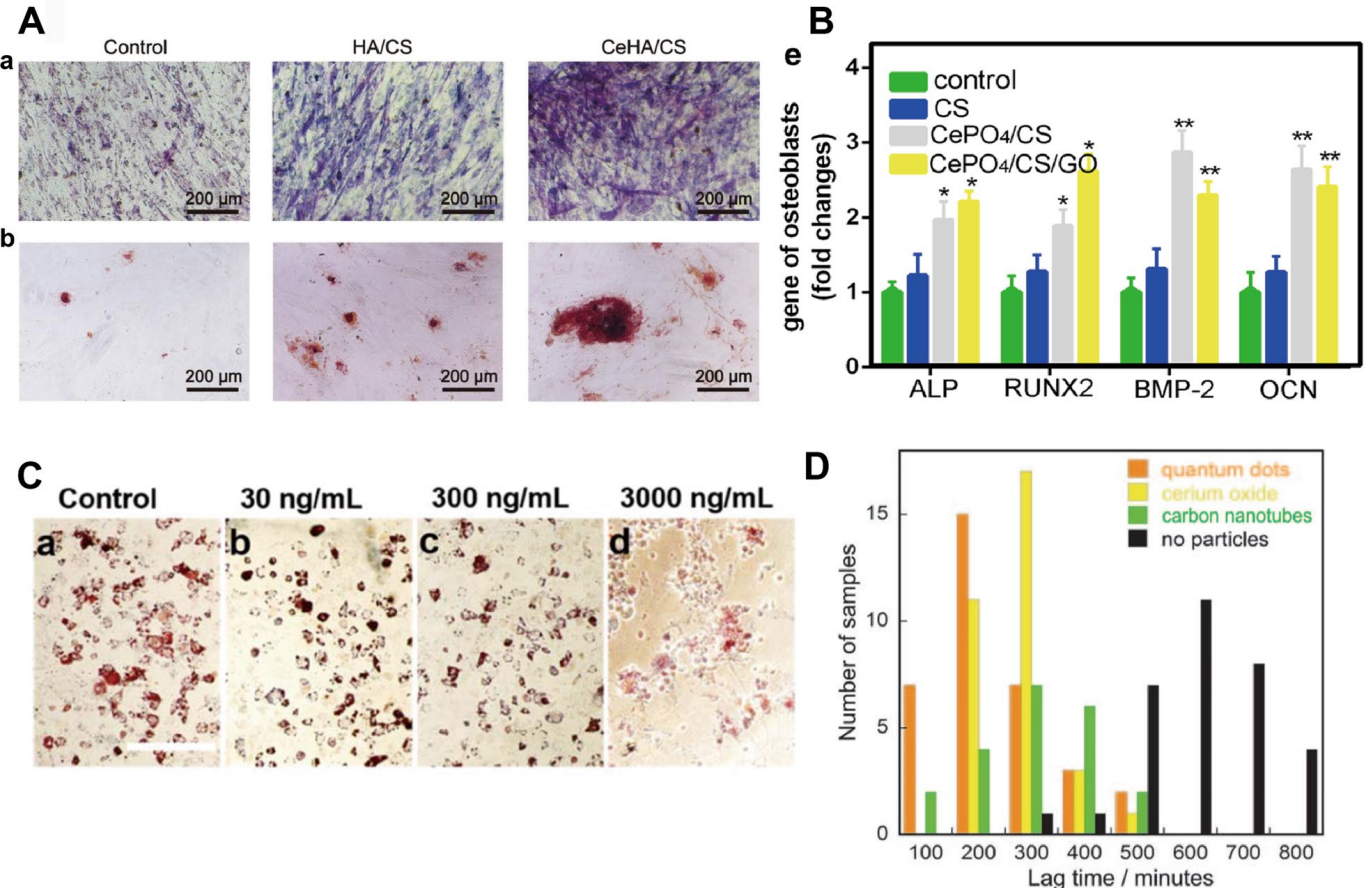
RE NMs promote osteogenesis in vitro. **A** (a) ALP staining and (b) alizarin red staining images of hBMSCs cultivated with control medium and extraction solution of HA/CS and CeHA/CS scaffolds for 7 and 20 days. Source: Reprinted with permission from ref. [164]. **B** RT‑PCR analysis of ALP, RUNX2, BMP‑2 and OCN expression in MC3T3‑E1 cells. Source: Reprinted with permission from ref. [8]. **C** Adipogenic differentiation of the rBMSCs after 7 days of treatment with NaYF4: Yb/Er at different concentrations. Source: Reprinted from ref. [189]. Copyright 2024, with permission from Elsevier. **D** Nucleation of collagen fibrillation by nanoparticles; CeO NPs, yellow. Source: Reprinted with permission from ref. [151]. HA, hydroxyapatite; CS, chitosan; CeHA/CS, nacre-mimetic cerium-doped layered hydroxyapatite/chitosan; GO, Graphene oxide; ALP, Alkaline Phosphatase; RUNX2, runt-related transcription factor 2; BMP-2, bone morphogenetic protein2; OCN, osteocalcin

**Fig. 4:**
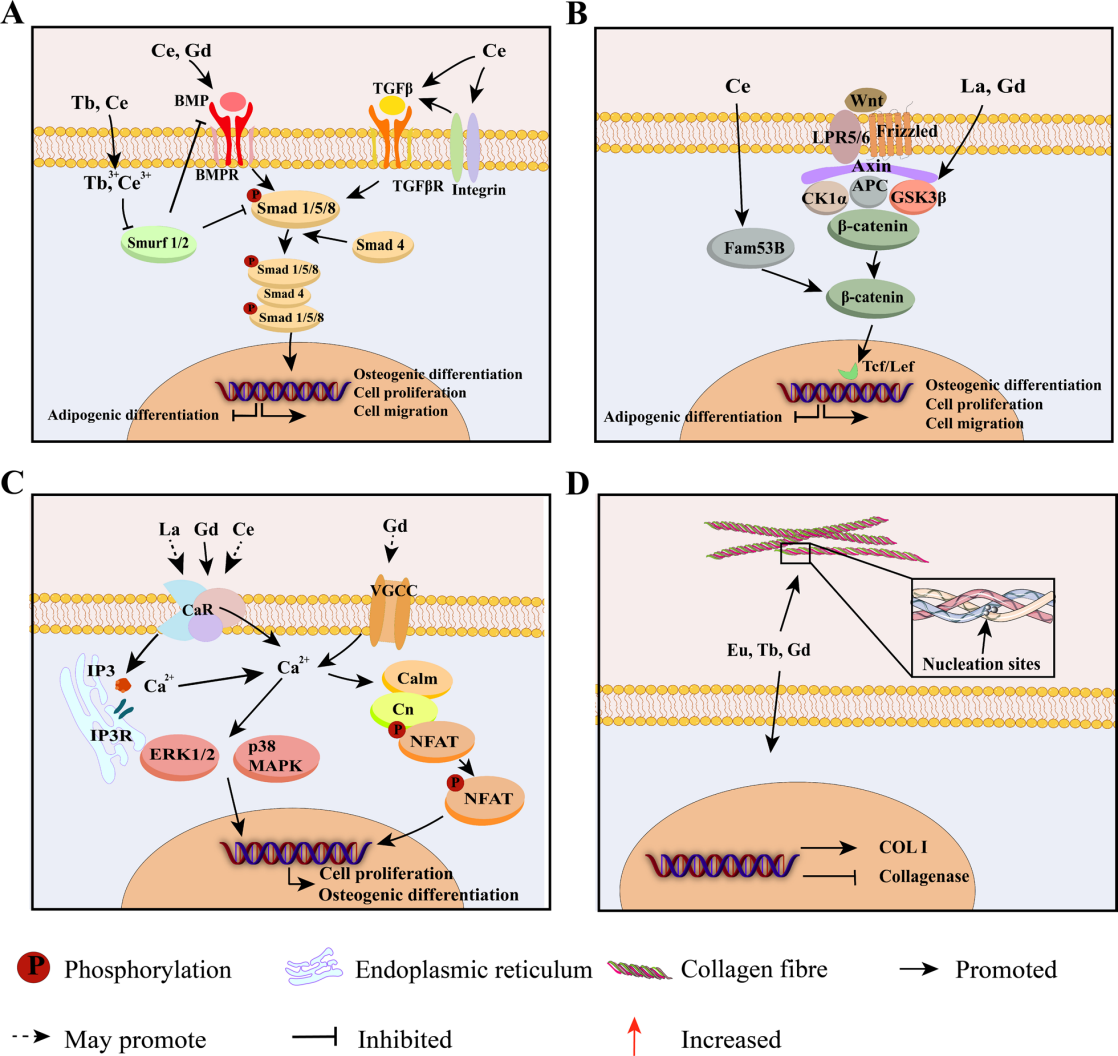
Direct osteogenic effects and mechanisms of RE NMs. RE NMs activate the TGF-β/BMP/Smad (**A**) and Wnt/β-catenin signalling pathways (**B**) to promote osteogenic differentiation, cell proliferation and migration and inhibit the lipogenic differentiation of MSCs. **C** RE NMs activate Ca^2+^ channels, increase intracellular Ca^2+^ levels, and promote cell proliferation and osteogenic differentiation. **D** RE NMs promote collagen secretion and the formation of collagen nucleation sites to promote collagen calcification. BMP, bone morphogenetic protein; BMPR, bone morphogenetic protein receptor; TGF β, transforming growth factor-β; Smad, small mothers against decapentaplegic; Wnt, wingless-type MMTV integration site family; CaR, calcium-sensitive receptors; VGCC, voltage-gated calcium channels; Calm, calmodulin; Cn, calcineurin; IP, inositol triphosphate; IPR inositol triphosphate receptor; COL I, type I collagen

MSCs can differentiate into many distinct mesenchymal cell types, such as osteoblasts, chondrocytes and adipocytes [126]. The osteogenic differentiation of bone marrow MSCs is a critical step in osteogenesis. RE NMs accelerated bone tissue formation promote MSC differentiation. It is reported that cerium oxide nanoparticles-modified bioglass (Ce-BG) scaffolds rapidly induced the growth of new osseous tissues and had positive effects on alkaline phosphatase (ALP) (an early phenotypic marker of osteogenesis) activity [127]. In addition, RE NMs promoted high expression of ALP, runt-related transcription factor 2 (RUNX2), osteopontin (OPN), bone sialoprotein II (BSP II) and osteocalcin (OCN) in MSCs [11, 12, 14, 65, 113, 127–129] (Fig. 3). Some RE NMs, such as NaGdF_4_:Yb/Er NPs [12] and NaYF_4_:Yb/Er nanocrystals [11], have also been shown to inhibit adipogenic differentiation [11, 12, 22], which can indirectly increase the differentiation of MSCs into osteoblasts [130] (Fig. 3C). This process is accompanied by the activation of the classical transforming growth factor-beta (TGF-β)/bone morphogenic protein (BMP)/Smad and wingless-INT (Wnt)/β-catenin signalling pathways. Additionally, they can activate Ca^2+^ channels and exert a significant influence on bone formation (Fig. 4).

##### TGF-β/BMP/Smad signalling pathway

TGF-βs and BMPs, which act on a tetrameric receptor complex, transduce signals to the canonical Smad-dependent signalling pathway to regulate osteogenic differentiation, bone formation and bone homeostasis [131]. RE NMs, including graphene-modified CePO_4_ nanorods [8], Eu-doped Gd_2_O_3_ nanotubes [113] and [Gd@C_82_(OH)_22_]n NPs [22], can activate the BMP signalling pathway and thereby promote osteogenic differentiation (Fig. 4A). They activate the TGF-β/BMP/Smad signalling pathway by interacting with BMP receptors (BMPRs) on the cell membrane [132], activating integrin-mediated TGF-β signalling [133] or indirectly increasing BMP expression. Smad1/5/8 are then phosphorylated and regulate the expression of genes related to osteogenic differentiation. The ability of RE NMs to inhibit adipogenic differentiation and thereby promote osteogenesis may also be attributed to the BMP/Smad1/5 signalling pathway [22]. This process may involve the downregulation of the adipogenic differentiation-related transcription factors C/EBP-α and PPARγ [132, 134]. Additionally, CeO NPs can increase endochondral osteogenesis, thereby promoting angiogenesis and facilitating bone regeneration [114]. This effect is potentially mediated by BMP2, which has the inherent capability to induce chondrogenic differentiation and stimulate endochondral bone formation [135].

In addition, the Smad ubiquitination regulatory factors Smurf 1 and Smurf 2 regulate BMP signalling via ubiquitination, thereby preventing excessive activation of TGF-β/BMP signalling [136]. Ce^3+^ and Tb^3+^ [124, 134] reduce the expression of Smurf 1 and Smurf 2 while inhibiting the subsequent degradation of Smad and BMP. This may enable RE NMs to further activate the TGF-β/BMP/Smad signalling pathway to promote osteogenic differentiation and inhibit adipogenic differentiation.

##### Wnt/β-catenin signalling pathway

The Wnt/β-catenin signalling pathway is extensively involved in fundamental processes of bone metabolism, including osteoblast proliferation, differentiation, and apoptosis [137]. The Wnt/β-catenin pathway suppresses the expression and transactivation of PPARγ mRNA by inducing histone H3 lysine 9 (H3K9) methylation on the target gene, thereby inhibiting MSC adipogenic differentiation [138].

According to previous reports, RE NMs, including La-LDH nanohybrid scaffolds [14] and CeO NPs [139], can activate the Wnt/β-catenin signalling pathway, thereby promoting the proliferation and osteogenic differentiation of MSCs. However, these materials activate the pathway in different ways. La-LDH nanohybrid scaffolds increase p-GSK-3β levels and promote the accumulation of β-catenin [14], while CeO NPs activate the Wnt pathway by facilitating the nuclear translocation of β-catenin through Fam53B [139]. In addition, Gd-based nanomaterials may promote the activation of the Wnt/β-catenin signalling pathway by a mechanism similar to that of La-based nanomaterials, as Gd^3+^ has been shown to upregulate Akt/GSK3β expression [140], which increases osteogenic capacity via the Wnt/β-catenin signalling pathway [141] (Fig. 4B).

The activation of the Wnt/β-catenin pathway by RE NMs promotes cell proliferation and osteogenic differentiation, thereby accelerating bone regeneration. However, whether RE NMs inhibit the adipogenic differentiation of MSCs through this signalling pathway and further promote osteogenic differentiation requires further study.

##### Activation of calcium channels

Cytosolic Ca^2+^ homeostasis is essential for multiple physiological functions, such as stem cell viability, cell proliferation and osteogenic differentiation [142]. RE NMs promote osteogenic differentiation by increasing intracellular Ca^2+^, mainly through the activation of CaRs and VGCCs, which is attributed to their calcium-mimicking and electrical properties (Fig. 4C).

The dissolution and degradation of RE NMs produce RE ions, which mimic Ca^2+^ and simulate CaRs, leading to an increase in intracellular Ca^2+^. Gd^3+^, for instance, activates ERK1/2 and p38 MAPKs and promotes osteogenesis via CaRs [33]. Similarly, La^3+^ has been shown to promote osteogenic differentiation by activating the ERK1/2 signalling pathway through the increase in intracellular Ca^2+^ [143]. The mechanism involves pertussis toxin (PTx)-sensitive Gi protein signalling [143], which indicates that these proteins are associated with Gi protein-coupled CaRs [144]. The activation of G protein-coupled receptors generates inositol triphosphate (IP3), which, upon binding to inositol triphosphate receptors (IP3Rs) in the ER, leads to ER Ca^2+^ release and potentiates the SOCE mechanism [142], thereby further promoting osteogenesis. Furthermore, CeO NPs have been reported to activate osteogenic differentiation by upregulating the ERK1/2 [127] and DHX15/p38 MAPK [114] pathways. This effect may be achieved through the activation of CaRs.

RE NMs have excellent electrical properties. Gd-doped barium titanate nanoparticles (Gd-BTO NPs) generate a negative surface potential and can cause oscillation of intracellular Ca^2+^ concentrations via VGCCs, activating the calcineurin (Cn)/nuclear factor of activated T cells (NFAT) signalling pathway and promoting osteogenic differentiation [40, 145]. However, studies have demonstrated that the majority of RE ions block VGCCs, which is the opposite of the effect of Gd-BTO NPs. In fact, the blocking effect of RE ions on VGCCs is voltage independent, as the mechanism involves occlusion of the channel pore through the binding of RE ions to a Ca^2+^/M^3+^ binding site [35]. Since Gd-BTO NPs are applied as nanocomposites, their release of Gd^3+^ is slower and less abundant than that of GdCl_3_. This finding increases the likelihood that VGCCs will be activated by electrical sites.

Therefore, when applying RE NMs in osteogenesis, it is crucial to consider the distinct effects of RE ions on different Ca^2+^ channels, as well as the diverse impacts of both RE NMs with electrical properties and different RE ions on Ca^2+^ channels.

#### Promotion of collagen secretion and calcification

Bone protein consists of 85% to 90% collagen. The collagen matrix plays a critical role in bone mineral deposition [146]. RE NMs can promote collagen secretion and calcification in vivo and in vitro. For instance, the La-[14] and Ce-[127] doped scaffolds were observed to augment the formation of both collagenous and non-collagenous organic matrix, as well as accelerate the deposition of collagenous fibers. The bone matrix consists mainly of type I collagen (COL I) [146]. COL I promotes osteogenic differentiation [147], and COL II promotes chondrogenic differentiation [148]. Eu-[116, 129], Gd-[10], and Tb-[129] doped nanomaterials significantly increased COL I secretion. Gd-[10] doped nanobunches promoted the secretion of COL I and COL II, which mediated osteogenic differentiation [147] and chondrogenesis [10].

Previous studies have shown that RE NMs can ensure stable collagen production by inhibiting collagenase activity [149] and decreasing the proteolytic sensitivity of collagen [150]. In addition, RE NMs promote collagen calcification in MSCs [22] and osteoblasts [25]. The porous structures significantly enhance the surface areas of RE NMs, thereby providing numerous functional groups on their surfaces. These functional groups serve as active sites for in vivo deposition of apatite and collagen, such as OH and O–Si–O [127]. They can form nucleation sites for protein fibrillation [151] **(**Fig. 3D**)**, increase the rate of collagen polymerization [152], stabilize collagen structure [153], and increase the strength of collagen structure [154] (Fig. 4D). RE NMs can effectively induce collagen secretion and calcification, creating a favourable microenvironment for tissue and bone regeneration [150, 155].

### Indirect osteogenic effects and mechanisms

In addition to the direct regulation of MSCs and osteoblasts, many immune cells in the bone microenvironment, such as macrophages, participate in immune regulation, and many newly formed blood vessels provide nutrition to new bone. This indirect regulatory effect can also effectively promote bone formation. Osteogenesis can be promoted indirectly by eliminating adverse factors, for example, inhibiting OC formation to limit bone absorption, regulating inflammatory responses to provide a suitable microenvironment for osteogenesis, and promoting tissue vascularization.

#### Inhibition of osteoclastogenesis and reduction in mature osteoclasts (mOCs)

**Fig. 5:**
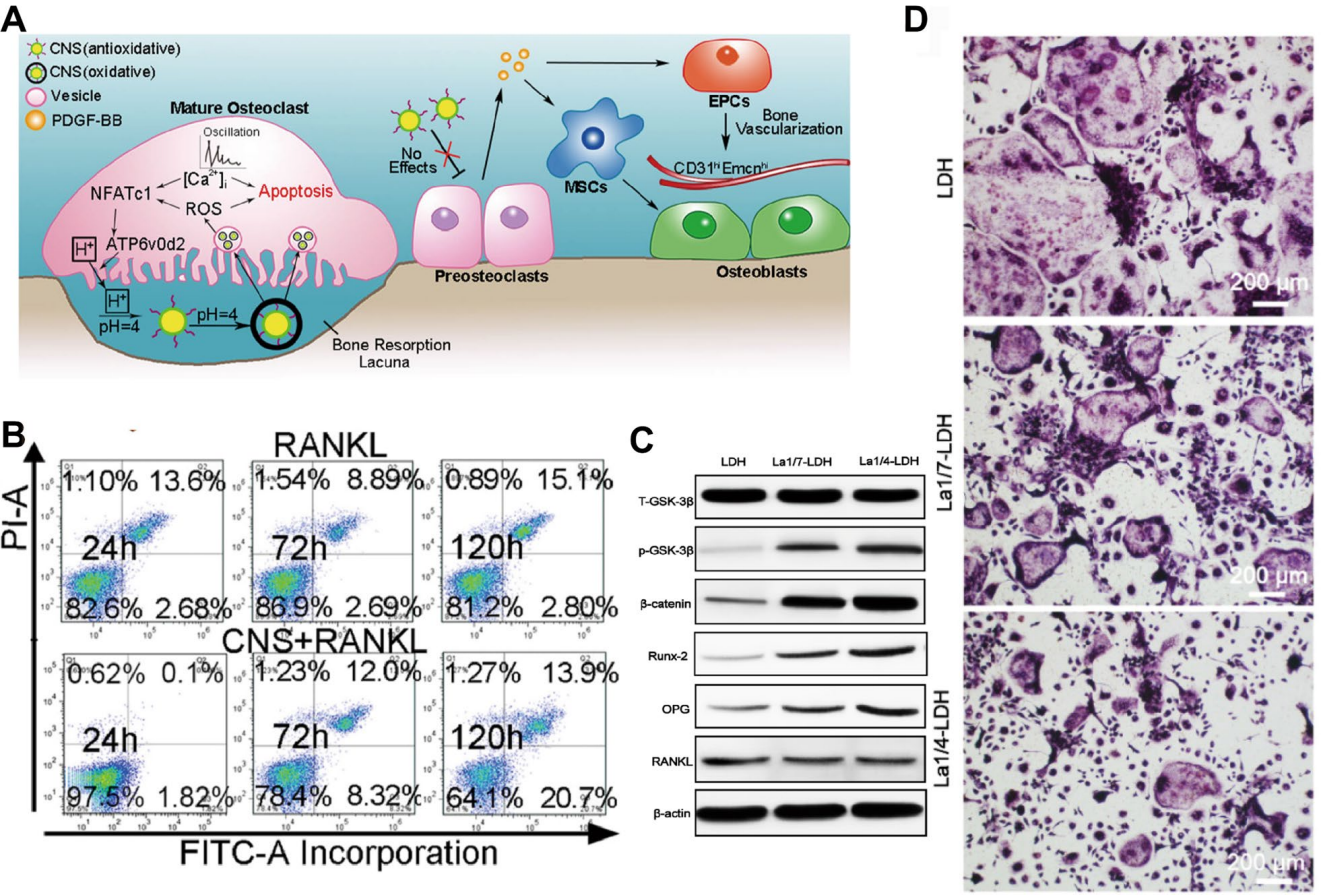
RE NMs inhibit osteoclasts to promote osteogenesis. **A** Schematic diagram of the mechanism through which CNS functions as a pro-anabolic therapy in OVX mice. **B** Annexin-V/PI staining was analysed via FCM to quantify the percentage of apoptotic early BMMs. Source: Reprinted with permission from ref. [157]. Copyright 2024, with permission from Elsevier. **C** Western blot results for t-GSK-3β, p-GSK-3β, β-catenin, Runx-2, OPG and RANKL expression in rBMSCs-OVX cultured with La-LDH and LDH scaffolds for 14 days. β-actin was used as an internal reference. **D** TRAP staining images of bone marrow macrophages cells cultured with La-LDH and LDH scaffolds in the presence of M-CSF (30 ng/mL) and RANKL (50 ng/mL) for 7 days. Source: Reprinted with permission from ref. [14]. Copyright 2024, with permission from Ivyspring International. CNS, cerium nano-system; RANKL, receptor activator of nuclear factor-κ B Ligand; GSK-3β, glycogen synthase kinase-3β; RUNX2, runt-related transcription factor 2; OPG, osteoprotegerin; La/LDH, lanthanum-substituted MgAl layered double hydroxide

Maintaining balance between bone formation and bone resorption is essential in bone metabolism [156]. Excessive OC activation causes bone loss and hinders bone regeneration. Accordingly, decreasing osteoclastogenesis or osteoclast function is a task of interest in bone regeneration research. RE NMs can block osteoclast-mediated bone resorption by inhibiting osteoclastogenesis [14, 25] and destroying mature osteoclasts (mOCs) [157, 158], thereby reducing bone loss and promoting osteogenesis (Fig. 5).

### Inhibition of osteoclastogenesis

**Fig. 6:**
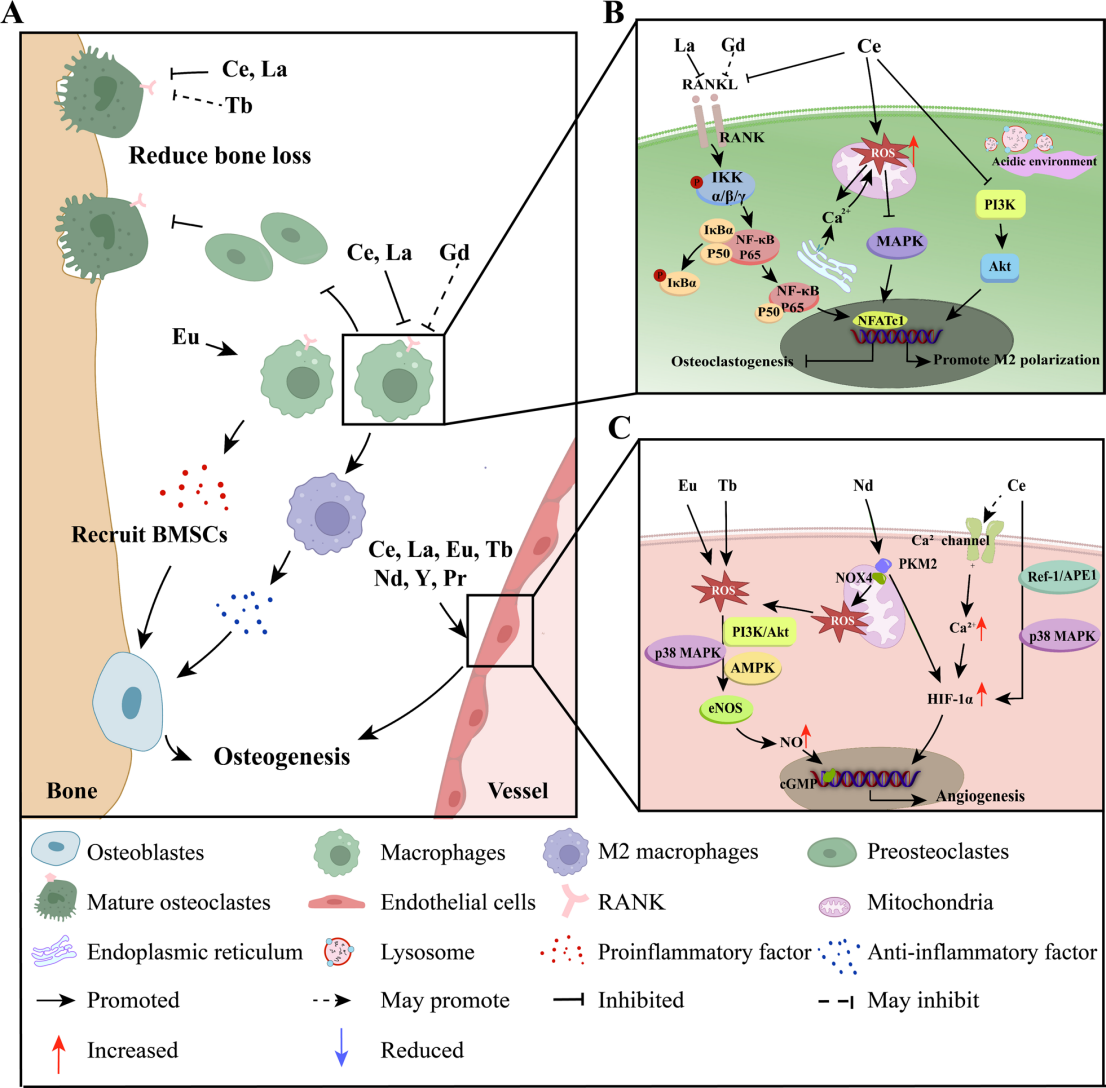
Indirect osteogenic effects and mechanisms of RE NMs. **A** RE NMs indirectly promote osteogenesis by regulating macrophages and endothelial cells. **B** RE NMs inhibit osteoclast differentiation through the NF-κB and MAPK signaling pathways in macrophages and may promote M2 polarization through the PI3K/Akt and MAPK signaling pathways to promote bone repair. **C** RE NMs induce hypoxia in ECs, leading to the upregulation of HIF1α and ROS and promoting angiogenesis and bone formation. HIF1α, hypoxia-inducible factor 1 alpha; ROS, reactive oxygen species

Within the bone marrow, macrophages proliferate and fuse into giant multinucleated mOCs, which are responsible for bone resorption [159]. This process involves several stages of differentiation, including preosteoclasts (pOCs), fused multinucleated osteoclasts, and ultimately mOCs [160]. Receptor activator of nuclear factor kappa-B ligand (RANKL) is a homotrimeric transmembrane protein [161]. Receptor activator of nuclear kappa-B (RANK) binds to RANKL and subsequently promotes osteoclastogenesis. This process involves activation of the NF-κB pathway, leading to the expression of NFATc1 and c-Fos, which play crucial roles in the regulation of osteoclast fusion [14, 162]. Osteoprotegerin (OPG) is a competitive inhibitor of RANKL. The OPG/RANKL ratio critically affects osteoclastogenesis [163]. RE NMs (*e.g.,* La-LDH nanohybrids [14] and CeO NPs [70, 78, 164]) were found to decrease RANKL expression and increase the OPG/RANKL ratio in MSCs, thereby suppressing RANKL-induced osteoclastogenesis (Fig. 5). In addition, Gd^3+^ increased the OPG/RANKL ratio in murine osteocytes [165], indicating that Gd-based nanomaterials may prevent bone loss by a similar mechanism to that of La-LDH nanohybrids and CeO NPs (Fig. 6B).

Moreover, CeO NPs have been reported to immediately acquire oxidase activity at pH = 4.0 [166] due to the inhibition of redox cycling from Ce^3+^ to Ce^4+^ by protons [167]. mOCs or macrophages then show a high number of lysosomes and an increase in ATPase H^+^-transporting V0 subunit D2 (ATP6v0d2), and the pH of the resorption lacuna reaches 3–4 [166]. Excessive ROS produced by CeO NPs can inhibit the NF-κB and MAPK signalling pathway-mediated activation of osteoclastogenesis [158].

### Reduction in mOCs

CeO NPs dose-dependently increased intracellular ROS levels in mOCs, but excessive ROS may decrease the resorptive function [158] and lead to direct cell destruction [112]. Excessive ROS can further sensitize endoplasmic reticulum (ER)-based Ca^2+^ channels, leading to the release of Ca^2+^ from the ER and increasing the concentration of Ca^2+^ in inner mitochondria [157, 168], which results in the uncontrolled release of both Ca^2+^ and ROS [169] (Fig. 6B). Consequently, significant cellular structural damage occurs, along with eventual apoptosis [158]. Tb [170] may have the same effect, as it acts as an oxidase within acidic bacteria. Notably, this oxidase activity of RE NMs does not affect pOCs [157], which promotes H-vessel formation and angiogenic–osteogenic coupling. This lack of effect on pOCs may be due to the relatively neutral pH in the cellular microenvironment.

#### Regulation of the immune microenvironment

The immune response associated with bone healing consists of an early acute inflammatory phase and a longer repair phase, and the transition is regulated mainly by macrophages [76]. In the acute inflammatory phase, the immune response is activated, leading to the secretion of inflammatory cytokines and chemokines by M1 (proinflammatory) macrophages to recruit MSCs. Upon polarization to the M2 phenotype (anti-inflammatory), these macrophages secrete anti-inflammatory cytokines along with osteogenic cytokines such as BMP2 and TGF-β [171, 172], thereby promoting new bone formation [76, 173].

**Fig. 7:**
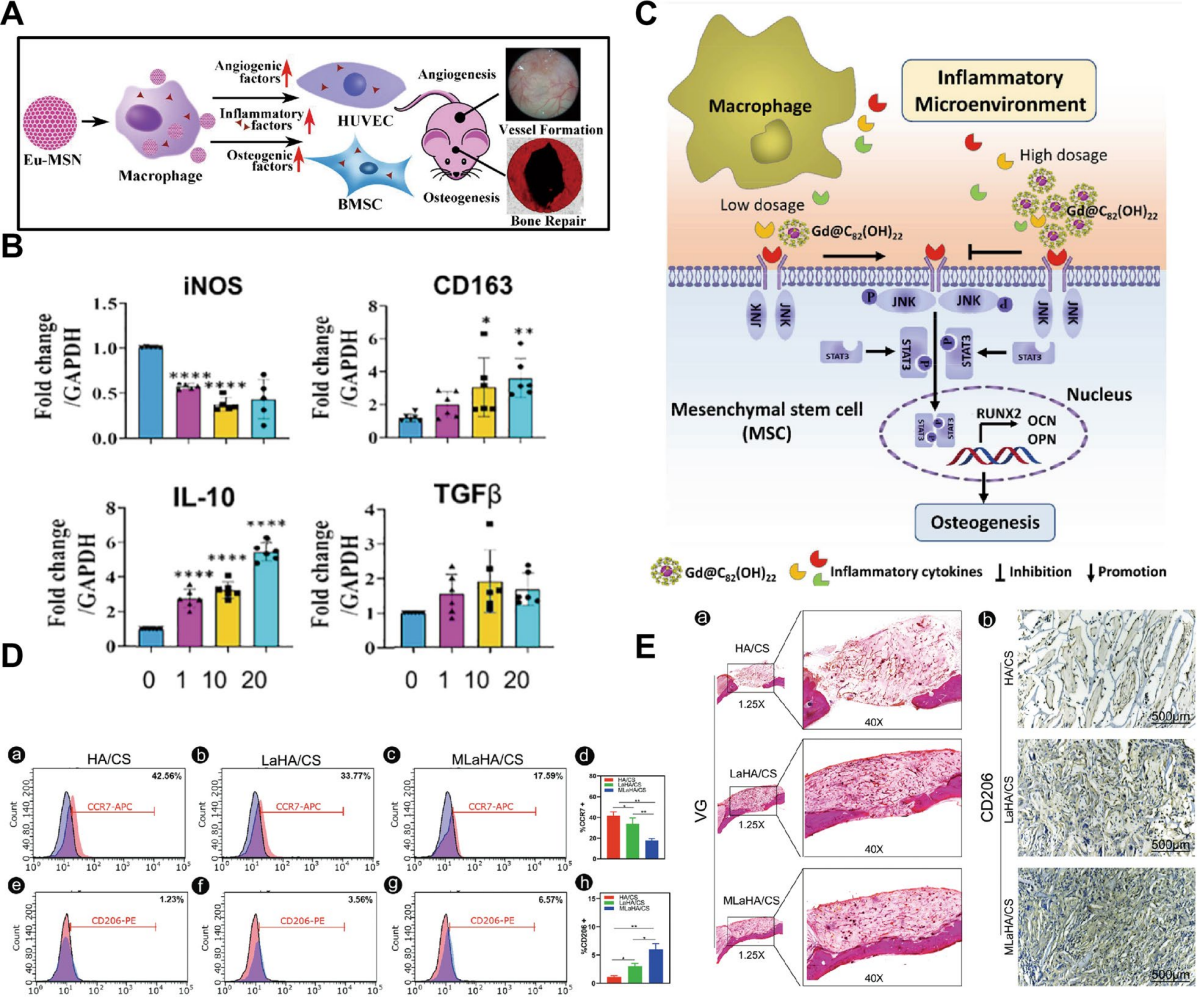
RE NMs regulate the immune environment to promote osteogenesis. **A** The prepared Eu-MSNs stimulated the polarization of macrophages to the inflammatory form, which further induced the osteogenic differentiation of BMSCs and induced the angiogenic differentiation of HUVECs. Source: Reprinted with permission from ref. [175]. Copyright 2024, with permission from Elsevier. **B** qRT‒PCR analysis of the gene expression of pro- and anti-inflammatory markers in 0, 1, 10 and 20 μg/mL CeO NPs after 3 days. Source: Reprinted with permission from ref. [9]. Copyright 2024, with permission from Elsevier. **C** Scheme of the mechanism by which Gd@C_82_(OH)_22_ modulates the osteogenesis of hMSCs through the JNK/STAT3 signalling pathway in the inflammatory microenvironment. Source: Reprinted with permission from ref. [65]. Republished with permission of Royal Society of Chemistry, 2024, permission conveyed through Copyright Clearance Center, Inc. **D** (a–h) Detection of M1 and M2 polarization by flow cytometry with macrophages labelled according to CCR6 and CD206 expression. Source: Reprinted with permission from ref. [23]. **E** (a) Van Gieson staining. (b) CD206 immunohistochemical staining of craniums with three cranial defects implanted with HA/CS, LaHA/CS, or MLaHA/CS scaffolds. Source: Reprinted from ref. [23] with permission of Royal Society of Chemistry, 2024, permission conveyed through Copyright Clearance Center, Inc. Eu-MSN, europium-doped mesoporous silica nanospheres; iNOS, inducible nitric oxide synthase; CD163, cluster of differentiation 163; IL-10, Interleukin-10; TGF β, transforming growth factor-β; CCR7, Recombinant Chemokine C–C-Motif Receptor 7; CD206, Macrophage mannose receptor 1; LaHA/CS, lanthanum-doped hydroxyapatite (HA)/chitosan (CS); MLaHA/CS, magnetic M-type hexagonal ferrite (SrFe_12_O_19_) nanoparticles incorporated LaHA/CS

RE NMs generate an appropriate immune response in macrophages [174, 175] and increase macrophage expression of osteogenic and angiogenic factors [175]. For example, Eu-doped mesoporous silica nanospheres (Eu-MSNs) (Fig. 7A) [175] modulate osteoimmunology and can enable vascularized osseointegration in bone regeneration. Moreover, Gd@C_82_(OH)_22_ modulates the inflammation-induced differentiation of MSCs through the c-Jun N-terminal kinase (JNK)/transcription 3 (STAT3) pathway [65] (Fig. 7C). The immunomodulatory effect of RE NMs helps promote stem osteogenic differentiation and increase the therapeutic efficacy of stem cell-based agents for biomedical regeneration in an inflammatory microenvironment.

On the other hand, the anti-inflammatory activity of RE NMs offers significant advantages in bone regeneration. These compounds reduce the levels of inflammatory factors, such as iNOS, within the bone microenvironment and facilitate the M2 polarization of macrophages [15]. CeO NPs [9, 15, 75], MLaHA/CS nanoscaffolds [23] and hydrated GdPO_4_ nanorods [7] have been reported to induce the M2 switch in macrophages (Fig. 6A, B). The mechanism involves inhibition of the PI3K-AKT signalling axis [15]. One study demonstrated that the inhibition of M1 polarization in osteoarthritis synovial macrophages by nintedanib is mediated by the MAPK/PI3K-AKT pathway, resulting in reduced articular cartilage degeneration [176]. The inhibitory effect of CeO NPs on ROS-induced MAPK production in macrophages may also contribute to the M2 polarization of macrophages. In addition, the effects of RE NMs may occur in part by activating CaRs and thus increasing sensitivity to Ca^2+^ [144]. Ca^2+^ can promote CaR-mediated M2 macrophage polarization, leading to osteoinduction [177]. However, whether immune cells other than macrophages are involved in the immunomodulation of osteogenesis by RE NMs remains to be studied.

#### Promotion of angiogenesis

**Fig. 8:**
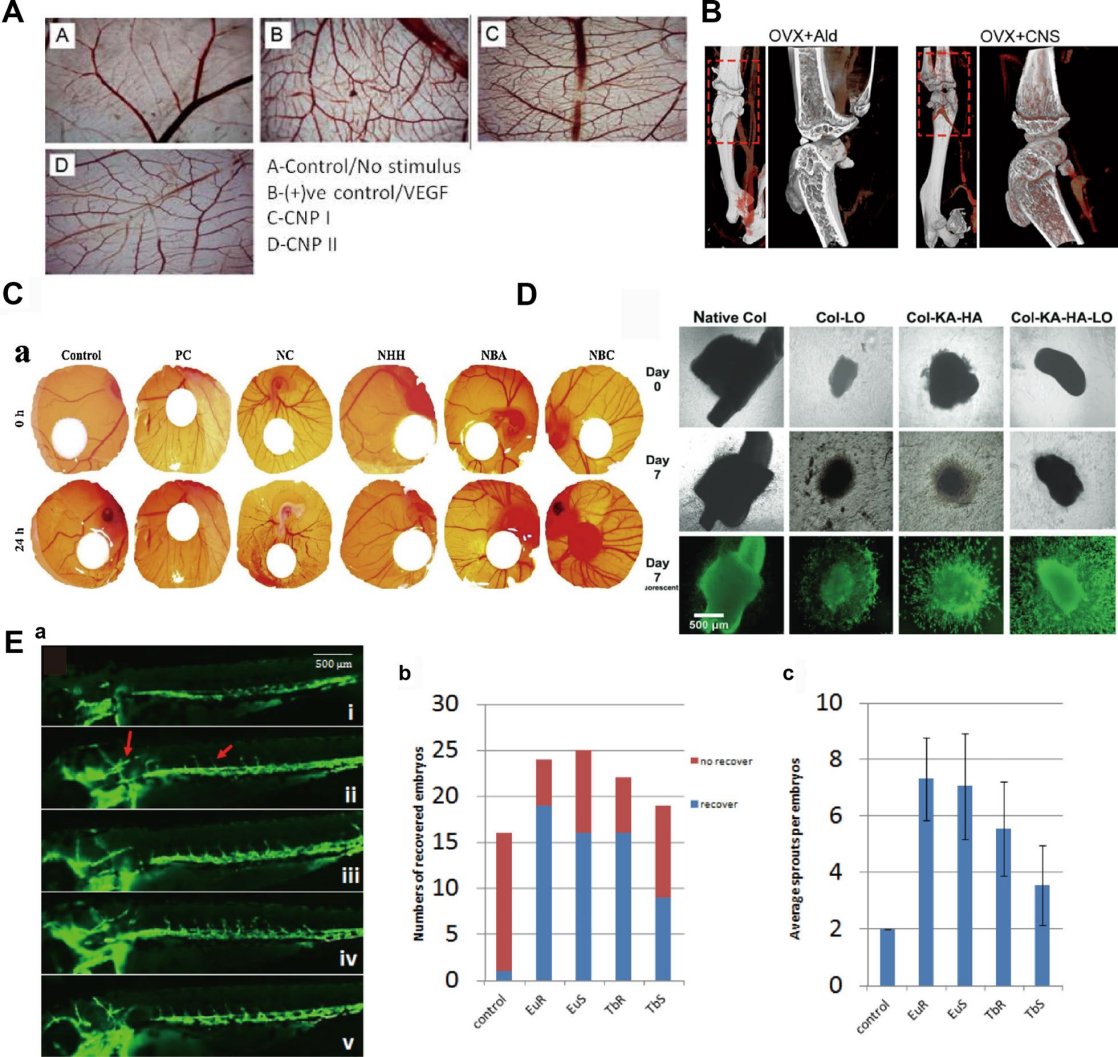
RE NMs promote angiogenesis to regulate osteogenesis. **A** CeO NP-induced angiogenesis was measured by a chick chorioallantoic membrane (CAM) assay (A–D). Source: Reprinted with permission from ref. [182], copyright 2024, with permission from Elsevier. **B** Microfil-perfused μCT angiography of OVX mouse femurs treated with Ald or CNS and quantification of vessel volume and surface area. Images are representative of 5 independent experiments. Source: Reprinted with permission from ref. [157]. **C** The in vivo angiogenic property of Nd nanopolymorphs was assessed by using a chick egg CAM model. Source: Reprinted from ref. [189], copyright 2024, with permission from Elsevier. **D** Microphotographs of the aortic arch sprout area. Source: Reprinted from ref. [13], copyright 2024, from WILEY. **E** (a) Zebrafish embryos at 72 h. (i) Blank control, (ii) 100 μg/mL Eu rods, (iii) 100 μg/mL Eu spheres, (iv) 100 μg/mL Tb rods, (v) 100 μg/mL Tb spheres. Compared with those in the blank control group, vessel sprouts were found in the ISV region and head under nanoparticle treatment. (b) Graph showing the numbers of ISV-recovered embryos. (c) Graph showing the average ISV sprouts per embryo. The number of ISV sprouts increased at different levels after nanoparticle treatment. Source: Reprinted from ref. [188], copyright 2024, with permission from WILEY. VEGF, vascular endothelial growth factor; CNP, cerium oxide nanoparticles; OVX, ovariectomized; Ald, alendronate; CNS, cerium nano-system; PC, positive control; NC, negative control; NHH, Nd nanoparticles, NBA, Nd nanocubes; NBC, Nd nanorod; Native Col, native collagen; Col-LO, collagen–lanthanum oxide; Col-KA, collagen-ƙ-carrageena; Col-KA-LO, collagen-ƙ-carrageenan-lanthanum oxide nanoparticle

**Fig. 9:**
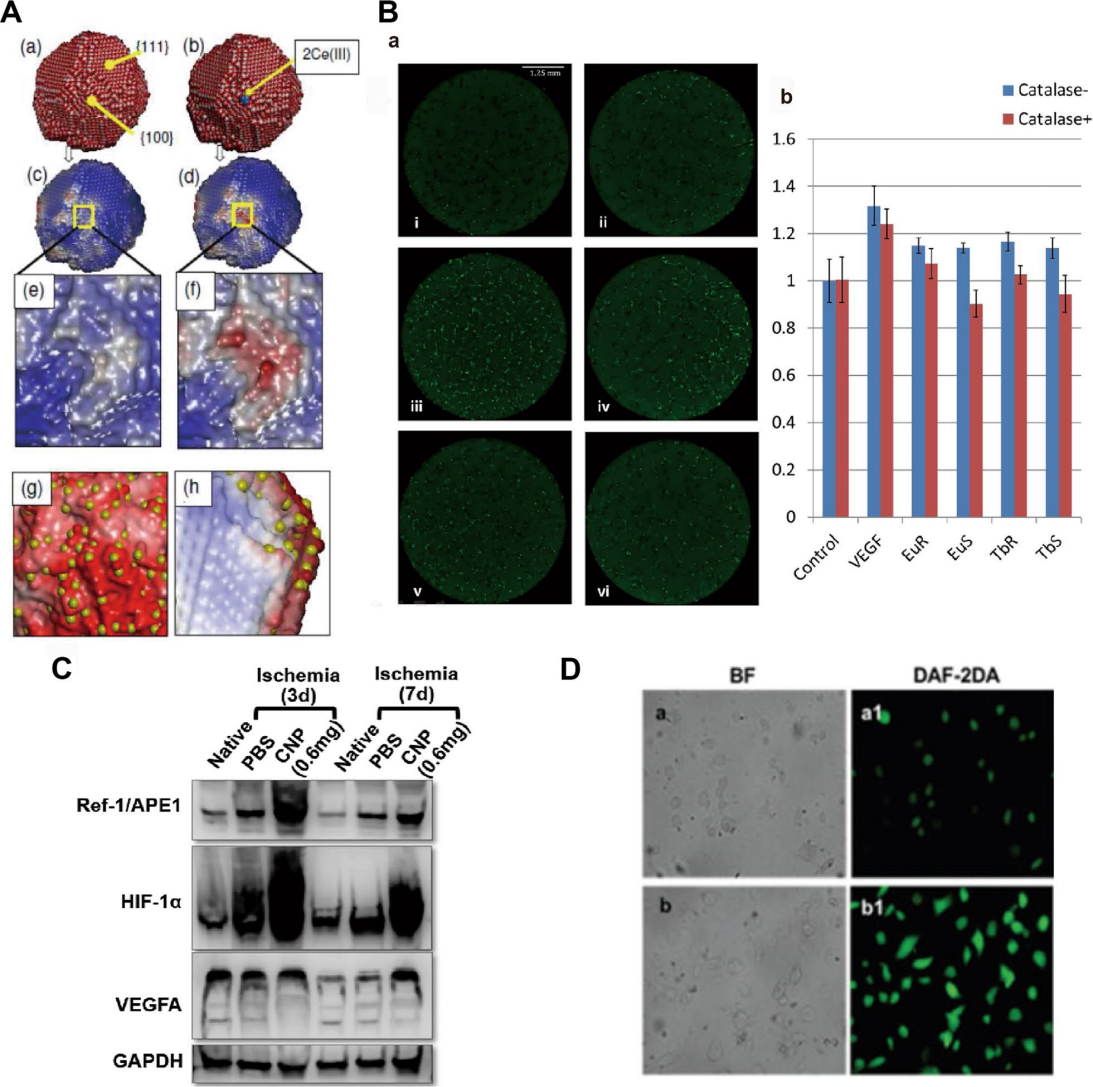
Mechanism by which RE NMs promote angiogenesis.**A** Atomistic models and (oxygen) electrostatic energy surfaces for CeO NPs. Sphere model representation of the atomic positions comprising (a) the unreduced CeO NPs and (b) the reduced CeO NPs. Oxygen is coloured red, Ce^4+^ is white, and Ce^3+^ is blue. (c) and (d) show the electrostatic energy surface maps of the unreduced and reduced CeO NPs, respectively, and enlarged views are shown in (e) and (f), respectively. (g) and (h) show an area of a CeO NP with a high concentration of Ce^3+^ (yellow spheres) on the surface. Domains near Ce^3+^ are red, indicating labile oxygen; conversely, domains relatively devoid of surface Ce^3+^ are blue, indicating reduced oxygen extraction reactivity. Source: Reprinted from ref. [182] , copyright 2024, with permission from Elsevier. **B** (a) Primary cell culture with 1 μg/mL nanoparticles and 1000 units/mL catalase. (i) Blank control, (ii) 1000 units/mL catalase, (iii) 20 ng/mL VEGF, (iv) 20 ng/mL VEGF and 1000 units/ml catalase, (v) 1 μg/mL Eu rods, (vi) 1 μg/mL Eu rods and 1000 units/mL catalase. (b) Quantitative analysis showing that catalase can abolish proangiogenic activities induced by nanoparticles but not by VEGF. Source: Reprinted from ref. [188] , copyright 2024, with permission from WILEY. **C** Western blot analysis showing greater levels of Ref-1/APE1, HIF-1α, and VEGFA in the 0.6 mg CNP group than in the PBS-only group at days 3 and 7. Source: Reprinted with permission from ref **D** Enhanced NO production by EHNs. Fluorescence imaging of nitric oxide production in EA: hy926 cells incubated with (a, a1) nothing (control) or (b, b1) EHNs (5 μg/mL). Source: Reprinted from ref with permission of the Royal Society of Chemistry, 2024; permission conveyed through the Copyright Clearance Center, Inc. Ref-1/APE1, apurinic/apyrimidimic endonuclease 1/ redox factor 1; HIF-1α, hypoxia-inducible factor 1 alpha; VEGFA, vascular endothelial growth factor A; CNP, cerium oxide nanoparticles; EHNs, europium hydroxide Eu(OH)_3_ nanorods

Increasing the number of capillaries can facilitate the delivery of growth factors, nutrients, and oxygen to expedite bone repair [4]. RE NMs can promote angiogenesis and enhance vascularized osteogenesis. On the one hand, the porous structures facilitate blood vessels ingrowth into the scaffold to provide nutrients to the nascent bone tissue [178]. Pore size, porosity, and pore interconnectivity [179] dictate the contact area of endothelial cells with the scaffold, which is promoted by mechanical signals to extend multiple thin filopodia increasing their adhesion and growth [180]. On the other hand, RE NMs induce macrophages to secrete anti-inflammatory cytokines and angiogenic factors (*e.g.,* CD31, MMP9 and VEGFR1/2) [175], which immunomodulate angiogenesis in the bone microenvironment. Angiogenesis can also be regulated by tissue-localized oxygen concentrations. RE NMs effectively promote angiogenesis by decreasing the intracellular oxygen concentration, prompting various cellular mechanisms to adapt to a low-oxygen environment (Figs. 8, 9).

Hypoxia-inducible factor-1α (HIF1α) is activated by hypoxia and serves as the key mediator of adaptation to hypoxia [181]. It can promote VEGF expression and thus the formation of new blood vessels to increase oxygen delivery [182]. RE NMs induce transient hypoxia in ECs, leading to the upregulation of HIF1α (Fig. 6A, C). *Das* [182] observed low O_2_ levels immediately after CeO NPs treatment for up to 1 h; however, O_2_ levels returned to normal after 2 h of CeO NPs treatment. The reason is that the oxygen vacancies in RE NMs can bind oxygen from inside the cell [182]. Additionally, CeO NPs not only increase the expression of HIF-1α but also stabilize HIF-1α through activating the Ca^2+^ channel of MSCs and increasing Ca^2+^ levels [183], highlighting their importance in angiogenesis regulation. Given the crucial role of Ca^2+^ in stabilizing HIF-1α within ECs [184–186], further investigation is warranted to explore whether other RE NMs can also induce angiogenesis in hypoxic endothelial cells by activating Ca^2+^ channels such as CaRs.

Another mechanism involved in the response to hypoxia is the formation of ROS [181]. They induce the activity of matrix proteases, which is one of the initial characteristics of angiogenesis and can provide space for EC migration. ROS can also interact with HIF1α to promote angiogenesis [178]. Some RE NMs (e.g., europium hydroxide [Eu(OH)_3_] nanorods [24, 187] and terbium hydroxide [Tb(OH)_3_] nanorods [188]) promote angiogenesis by releasing controlled amounts of ROS (primarily H_2_O_2_) into the cytoplasm [187, 188] (Fig. 9B). The ROS produced by Eu(OH)_3_ nanorods can also activate endothelial NOS (eNOS) in a PI3K/AMPK/Akt-dependent manner [24, 187, 189], which further promotes angiogenesis [178] (Fig. 9D). Nd nanopolymorphs achieved a combined angiogenic effect of increasing HIF-1α and ROS [189] (Fig. 6C).

RE NMs regulate HIF-1α and ROS-mediated angiogenesis effectively accelerates bone regeneration, especially the development of H-type vessels [114, 157, 190, 191], which participate in endochondral angiogenesis and osteogenesis [192]. Biocompatible cerium nano-system (CNS) also induced an increase in vessel volume and surface area of femurs in mice, as well as the formation of H-shaped blood vessels, resulting in a reduction of bone loss in vivo [157] (Fig. 8B). These findings suggest that RE NMs are an ideal material for vascularized bone regeneration.

## Factors that influence the effects of RE NMs

### Nanoforms of REEs

Here, we discuss the osteogenic properties of RE NMs, which exhibit advantages over RE ions in bone regeneration. First, RE NMs exhibit lower cytotoxicity [193] and possess larger surface areas for binding with bone tissue [194] than RE ions. The nanomechanical signals on the surfaces of RE NMs are transmitted to cells through integrins and mechanosensitive ion channels [173]. Aligned nanofibers also facilitate orderly cell arrangement, promote migration and stimulate cell proliferation during bone healing [195]. Second, because of their crystal structures contain oxygen vacancies (e.g., Eu-doped Y_2_O_3_ NPs [71]) and elemental valence transitions (e.g., CeO NPs [56]), RE NMs exhibit stronger antioxidant and angiogenic effects in bone regeneration. Third, RE NMs have been used in a wider range of applications in clinical settings. The rough and porous surface of RE NMs provides a disordered pore system that facilitates the loading and sustained release of osteogenic drugs [194]. RE NMs possess structural advantages that lack ions, making them more suitable for bone implants than either RE ions or micron-sized materials.

### Particle size

NP size has significant effects on the proliferation, differentiation, mineralization, and angiogenesis of osteoblasts [182]. The cellular response mediated by NPs is determined by their size, with a particle size of 40–60 nm exhibiting the greatest effect [22]. Studies have demonstrated that the impact of RE NMs on osteogenesis depends on their size [12, 196]. The uptake of NPs by cells plays a crucial role in determining osteogenesis, and size influences cellular uptake due to its influence on the enthalpic and entropic properties that govern the strength of adhesion between NPs and cellular receptors [197]. Specifically, 40 nm CeO NPs promote better osteogenic differentiation and mineralized matrix nodule formation than 60 nm particles [196]. This difference might be due to the higher surface-to-volume ratio of smaller RE NMs. In general, smaller CeO NP sizes are associated with higher surface Ce^3+^/Ce^4+^ ratios [198] and stronger osteogenic effects. However, NPs larger than 60 nm in diameter lead to receptor shortages, resulting in decreased uptake due to an increasing entropic penalty [199]. On the other hand, very small NPs cannot occupy multiple receptor binding sites before undergoing phagocytosis; they can, however, physically block pore structures in the plasma membrane, such as ion channels, and thus hinder ion exchange processes [200]. Furthermore, BMSCs were found to take up 30 nm βNaGdF_4_: Yb/Er nanocrystals more efficiently than 15 nm nanocrystals. This experiment demonstrated that larger βNaGdF_4_: Yb/Er nanocrystals promote osteogenic differentiation while slightly inhibiting adipogenic differentiation [12]. When utilizing RE NMs to promote bone formation, it is essential to design suitable NP sizes. Although the toxicity of NPs is lower than that of particles of other sizes, this toxicity should not be ignored in developing bone regeneration applications.

### Concentration and dose

The concentration and dose of RE NMs influence their biological effects, including their effects on the proliferation, differentiation, adipocyte transdifferentiation and mineralization of primary osteoblasts and BMSCs [196]. The osteogenic effects of these agents exhibit a “low-promotion, high-inhibition” hormesis pattern. In other words, a low concentration or dose of RE NMs promotes one formation by rBMSCs and inhibits lipogenesis, while a high concentration or dose inhibits bone formation [70, 141]. Furthermore, Gd@C_82_(OH)_22_ (< 1 μM) markedly upregulated the osteogenic differentiation of hMSCs. In contrast, a higher concentration (> 2 μM) of Gd@C_82_(OH)_22_ significantly suppressed osteogenesis, and 5 μM and 10 μM Gd@C_82_(OH)_22_ promoted the adipogenic differentiation of hMSCs [65]. NaYF_4_:Yb/Er nanocrystals [11], CeO NPs [9, 114] and Tb/MBG nanospheres [201] had similar effects. Due to the concentration-dependent effects of RE NMs on osteogenesis, the controlled release of RE NPs or RE ions is a challenge that must be considered when designing rare earth-doped materials. *Xu* [202] developed poly(lactide coglycolide) (PLGA)-based microsphere-based 3D porous scaffolds as La^3+^ storage and release systems to promote osteogenesis. RE NMs with sustained release properties are ideal bone implant materials.

### Surface topography

The surface topography is the most important feature of cell-modulating scaffolds, which control the early biological responses of cells, including adhesion, spreading and migration, and subsequently alter their phenotype to regulate bone regeneration [10]. An increase in the surface roughness of RE NMs can increase the efficiency of bone formation, matrix mineralization and calcium deposition [203]. The 3D pore structures [14, 127] and narrow mesopore size distribution [201] on the surfaces of RE NMs promote cell adhesion and pseudopodium migration and increase scaffold osteoconductivity. Hollow cores and mesopore shells provide additional active sites for bone formation [127]. In response to these morphological properties of material surfaces, stem cells and osteoblasts change shape and tension, modulate downstream pathways, and attach to nanofibers to promote bone formation [204]. The organization of CeO NPs within the biopolymer into self-assembled line-like patterns at multiple scales enables MSCs to grow in a way that aligns with the NP pattern [205]. The efficiency of bone regeneration can be further enhanced by designing and applying RE NMs with different surface morphologies.

### Others

The shape, surface modification, and valence state of REEs are also influential factors in the osteogenesis of RE NMs. (i) Manipulating the morphology of RE NMs can impact cellular uptake [189]. Short CeO NPs (NPs and nanorods) undergo rapid internalization by cells and suppress ROS production, whereas long CeO NPs (nanowires) exhibit slower internalization kinetics [206]. (ii) Surface modification plays a crucial role in increasing cell–material interactions while reducing material cytotoxicity [10]. Compared with materials without surface modifications, polymer PBLG-modified GdPO_4_·H_2_O nanobubbles significantly increase COL I and COL II expression levels by at least threefold, thus promoting bone regeneration [10]. iii) For CeO NPs, which are the most extensively studied among all RE NMs, the surface valence state of Ce regulates osteoblast activity and proliferation. Elevated levels of Ce^4+^ promote osteoblast proliferation, whereas increased concentrations of Ce^3+^ hinder MSC activity [207]. These observations may be attributed to the SOD and CAT mimetic activities of CeO NPs [208].

## Prospects and limitations

From a broad perspective, RE NMs may exhibit similar properties in the field of promoting bone regeneration, allowing us to gain insights into new RE NMs by comparison to other RE NMs that have been extensively studied in osteogenesis research, such as CeO NPs [17, 208]. Although not directly reported to promote osteogenesis, some RE NMs may improve the material properties of bone implants. For instance, Dy can optimize the mechanical strength and degradation rates of zinc-based alloys while conferring excellent antibacterial ability and cytocompatibility towards MC3T3-E1 cells [209]. We review the osteogenic properties of RE NMs and their direct or indirect regulatory effects on MSCs and osteoblasts, as well as macrophage- and endothelial cell-mediated osteogenesis. Nevertheless, numerous novel challenges have arisen.

### Design of novel RE NMs biocomposites

The future design of RE NMs for promoting osteogenesis will combine direct and indirect osteogenic effects to obtain multifunctional bio-nanocomposites with optimal regulatory effects on various cells within the osteogenic microenvironment. *Ge* [8] demonstrated a bioactive scaffold composed of graphene-modified CePO_4_ (CePO_4_/CS/GO) nanorods that promoted angiogenesis and macrophage polarization and induced bone formation by activating the BMP2/Smad signalling pathway. However, the fact is that most of the current studies on the bone immunomodulation by RE NMs focus on macrophages, as we mentioned above. Given the intricate nature of the immune system and the interplay among multiple immune cells, it is plausible that RE NMs may exert regulatory effects on various types of immune cells within the bone immune milieu. Studies have shown that RE NMs have regulatory effects on lymphocytes, monocytes [210], T cells and leukocytes [65]. Whether RE NMs regulate other immune cells in the osteogenic microenvironment is worthy of further discussion. Developing novel biocomposites of RE NMs that regulate multiple immune cells for potential osteogenesis applications will be the focus of future research.

In addition, the luminescence and magnetism of RE NMs can enable the visualization of bone implant [129]. For example, in vivo MRI and X-Ray bifunctional imaging of GdPO_4_·H_2_O nanobundles were designed for tracing bone implant and bone regeneration [128]. The utilization of RE NMs in the development of multifunctional biomaterials for osteogenesis holds significant potential.

### Toxicity of RE NMs for biological applications and potential solutions

Despite the low cytotoxicity of RE NMs, the toxicity caused by excessive deposition of them still requires special attention. RE NMs can enter the human body through inhalation, oral ingestion, and dermal contact [211–215]. They accumulate mainly in bone, liver, and spleen [29, 216, 217], giving rise to various toxic effects such as neurodegeneration [218, 219], damage to the reproductive system [220] and hemolysis [193].

The primary mechanism of toxicity for RE NMs can be summarized as follows: (i) Impairment of mitochondrial function. Prolonged exposure to RE NMs leads to mitochondrial dysfunction, resulting in the generation of ROS [218, 221]. Under acidic conditions, CeO NPs as oxidase also induce cytotoxicity by promoting ROS production. (ii) Disruption of lysosomal integrity. RE NMs with high aspect ratios can act as fiber-like substances that damage lysosomes. For instance, CeO nanorods cause progressive pro-inflammatory effects and cytotoxicity at lengths ≥ 200 nm and aspect ratios ≥ 22 [222]. Additionally, Y_2_O_3_ NPs dissolve and transform into YPO_4_ within acidifying intracellular lysosomes of BMSCs, leading to an imbalance in phosphate levels and inducing lysosomal- and mitochondrial-dependent apoptosis pathways [29]. (iii) Inhibition of Ca^2+^ channels in the cell membrane. RE ions possess properties similar to Ca^2+^, which disrupt normal cellular function by blocking Ca^2+^ channels and disturbing intracellular Ca^2+^ homeostasis [36, 37]. The neurotoxicity of La has received considerable attention due to its ability to block Ca^2+^ channels within the nervous system [223]. Similar to other metal nanoparticles, the toxicity of RE NMs is influenced by various factors, including ion release, synthesis method [224], particle size and shape [225], surface charge, cell type, dose and exposure route [226].

Previous studies have employed various methods to mitigate toxicity, including the design of biocomposite materials with sustained release properties [202] or of RE NMs coated with other materials [227]. For instance, PLGA-based microsphere-incorporated La-doped 3D porous scaffolds has demonstrated slow-release properties of La^3+^, reducing the toxicity of scaffolds and remaining within a safe range for 28 days [202]. Increasing the crystallinity of RE NMs can serve as an alternative approach for sustained release of RE ions [8]. Furthermore, the reduction of toxicity is facilitated by the coating or functionalization of RE NMs with other materials [10, 227]. Studies have shown that dextran-coated CeO NPs [207, 208] and polymer PBLG-functionalized GdPO_4_·H_2_O nanobunches [10] as effective methods for mitigating undesired effects and enhancing bioavailability. The phosphate imbalance of BMSCs induced by Y_2_O_3_ NPs can be effectively mitigated through the coating of YPO_4_ [29]. It is important to evaluate the accumulation and clearance mechanisms of RE NMs. Addressing these issues will aid in designing convenient and efficient RE NMs for clinical application.

## Conclusion

Because of their unique physicochemical properties and biological advantages, RE NMs have demonstrated significant potential for bone regeneration. They not only directly enhance bone regeneration but also modulate the immune microenvironment and promote angiogenesis, thereby indirectly facilitating osteogenesis. RE NMs effectively promote cell proliferation, adhesion, migration, and osteogenic differentiation. Additionally, they stimulate collagen secretion and deposition. Furthermore, RE NMs inhibit osteoclast formation, induce the M2 polarization of macrophages, and promote vascularization to establish a microenvironment that is conducive to bone regeneration. We discuss the factors influencing the osteogenic effects of RE NMs, as well as future research directions and their potential applications in bone regeneration. This review provides researchers with valuable insights into maximizing the utilization of RE NMs in osteogenesis.

## Data Availability

Not applicable.

## Abbreviations

Akt: Protein kinases B
ALP: Alkaline phosphatase
AMPK: AMP-activated protein kinase
ARE: Antioxidant response element
BMP: Bone morphogenetic protein
BMPR: Bone morphogenetic protein receptor
BMSCs: Bone marrow mesenchymal stem cells
BTE: Bone tissue engineering
Ca: Calcium
Calm: Calmodulin
CaR: Calcium-sensitive receptors
CAT: Catalase
CD31: Platelet endothelial cell adhesion molecule-1
Ce: Cerium
Cn: Calcineurin
CeONPs: Cerium Oxide Nanoparticles
COL I: Type I collagen
COL II: Type II collagen
ECM: Extracellular matrix
Eu: Europium
FA: Focal adhesion
Gd: Gadolinium
Gd-BTO NPs: Gd-doped barium titanate nanoparticles
GSK-3β: Glycogen synthase kinase-3β
HAp: Hydroxyapatite
HIF-1α: Hypoxia-inducible factor 1-α
IP3: Inositol triphosphate
IP3Rs: Inositol triphosphate receptors
JNK: C-Jun N-terminal kinase
La: Lanthanum
La-LDH: Lanthanum substituted layered double hydroxide
MLaHA/CS: Magnetic lanthanum-doped hydroxyapatite/chitosan
MSC: Mesenchymal stem cell
mTOR: Mammalian target of rapamycin
NADPH: Nicotinamide adenine dinucleotide phosphate
Nd: Neodymium
YAG: Yttrium aluminum garnet
NF-κB: Nuclear factor kappa-B
NOX: NADPH oxidase
Nrf2: Nuclear factor erythroid 2-related factor 2
pOCs: Preosteoclasts
Pr: Praseodymium
Pr_6_O_11_ NPs: Praseodymium oxide nanoparticle
RANK: Receptor activator of nuclear kappa-B
RANKL: Receptor activator of nuclear factor κB ligand
RE NPs: Rare earth nanoparticles
REEs: Rare earth elements
RE: Rare earth
ROS: Reactive oxygen species
RUNX2: Runt-related transcription factor 2
SACC: Stretch-activated calcium channel
SIRT1: Sirtuin 1
Smad: Small mothers against decapentaplegic
SOD: Superoxide dismutase
Tb: Terbium
TGF-β: Transforming growth factor-β
TRPC1: Transient receptor potential canonical channel 1
VEGF: Vascular endothelial growth factor
VEGF-R2: Vascular endothelial growth factor receptor 2
VGCC: Voltage-gated calcium channels
Wnt: Wingless-type MMTV integration site family
Y: Yttrium
